# Author Correction: Far-field phonon coupling in valley metamaterial circuits

**DOI:** 10.1038/s41467-026-71465-1

**Published:** 2026-04-27

**Authors:** Yao Huang, Weitao Yuan, Zhiwei Guo, Qi Wang, Yuxuan Zhang, Yueting Zhou, Yongdong Pan, Jia Zhou, Oliver B. Wright, Zheng Zhong, Jinfeng Zhao

**Affiliations:** 1 School of Aerospace Engineering and Applied Mechanics, Tongji University, Shanghai, 200092 China; 2 Sichuan Province Key Laboratory of Advanced Structural Materials Mechanical Behavior and Service Safety, School of Mechanics and Aerospace Engineering, Southwest Jiaotong University, Chengdu, Sichuan 611756 China; 3 MOE Key Laboratory of Advanced Micro-Structured Materials, School of Physics Science and Engineering, Tongji University, Shanghai, 200092 China; 4 School of Microelectronics, Nanjing University of Science and Technology, Nanjing, 210094 China; 5 State Key Laboratory of ASIC and System, School of Microelectronics, Fudan University, Shanghai, 200433 China; 6 Hokkaido University, Sapporo, Hokkaido 060-0808 Japan; 7 School of Science, Harbin Institute of Technology, Shenzhen, 518055 China

**Keywords:** Acoustics, Topological matter, Structural materials

Correction to: *Nature Communications* 10.1038/s41467-025-67108-6, published online 11 December 2025

Following publication of this article, the text has been clarified for notation and internal consistency. The changes do not affect the results or conclusions of the study. Changes have been made in the Results: “Valley metamaterial circuits: three configurations,” “Single-cavity far-field waveguide system,” “Dual-cavity waveguide system” and “Space-controlled sympathetic resonances” sections; Fig. 2c legend; Discussion (first paragraph); Methods: “Experimental Methods,” “Numerical calculations,” and “Theory of the waveguide-cavity dynamics” sections. For comparison, the original, uncorrected article is available as Supplementary Information accompanying this amendment. The changes are made in the HTML and PDF versions of the article.

## Supplementary information

Original, uncorrected article

## Supplementary information

Original, uncorrected article at 10.1038/s41467-026-71465-1

