# Supplementary material for: Far-field phonon coupling in valley metamaterial circuits
Source: Nat Commun. 2025 Dec 11;17:422. doi: 10.1038/s41467-025-67108-6 (PMC12796208; doi:10.1038/s41467-025-67108-6)
Supplement: Supplementary file 1 — Supplementary Information [file 41467_2025_67108_MOESM1_ESM.pdf]

Supplementary Information

Far-field phonon coupling in valley metamaterial circuits

Yao Huang[1], Weitao Yuan[2], Zhiwei Guo[3], Qi Wang[4], Yuxuan Zhang[1], Yueting Zhou[1], Yongdong Pan[1], Jia Zhou[5], Oliver B. Wright[6], Zheng Zhong[7], Jinfeng Zhao[1*]

[1] School of Aerospace Engineering and Applied Mechanics, Tongji University, 100 Zhangwu Road, Shanghai 200092, China

[2] Sichuan Province Key Laboratory of Advanced Structural Materials Mechanical Behavior and Service Safety, School of Mechanics and Aerospace Engineering, Southwest Jiaotong University, Chengdu, Sichuan 611756, China

[3] MOE Key Laboratory of Advanced Micro-Structured Materials, School of Physics Science and Engineering, Tongji University, Shanghai 200092, China

[4] School of Microelectronics, Nanjing University of Science and Technology, Nanjing 210094, China

[5] State Key Laboratory of ASIC and System, School of Microelectronics, Fudan University, Shanghai 200433, China

[6] Hokkaido University, Sapporo, Hokkaido 060-0808, Japan

[7] School of Science, Harbin Institute of Technology, Shenzhen 518055, China

*Corresponding author email: jinfeng.zhao@tongji.edu.cn

Supplementary Note 1. Sample parameters and experimental setup

Supplementary Table 1. Geometrical parameters of Samples 1–3

Sample	Type of cavity	Lattice constant a (mm)	Wafer thickness e_t (mm)	Pillar height h_p (µm)	Pillar Side length s_l (µm)
1	Single, far-field	641	525	292	500
2	Single, near-field	641	525	289	526
3	Dual (near + far)	641	525	289	526

Supplementary Table 2. The recipe for the BOSCH process

Process	Platen power (Watt)	Pressure (mTorr)	SF6 (sccm)	C4F8 (sccm)	Time (s)
Passivation	0	19	0	85	7
Etching	20	37	175	0	12

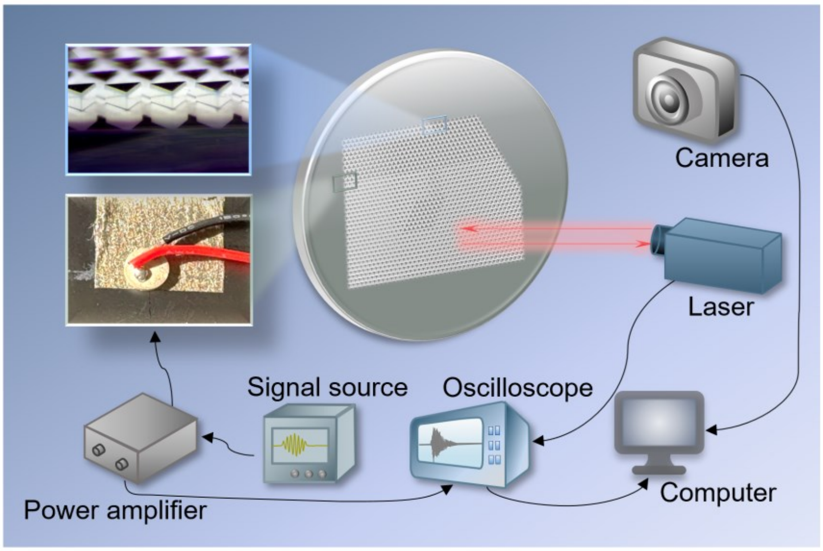

Supplementary Figure 1. Experimental piezo-laser setup for measuring ultrasonic wave fields in the cavity-waveguide circuit. Top left inset: an enlarged part of the sample (top middle panel). Middle left inset: a PZT disk bonded to the back surface of the sample. A seven-cycle burst signal centered at frequency f_c is generated by the signal source, amplified by the power amplifier, and delivered to the PZT disk. The laser measures the out-of-plane displacement (u_z) within target regions, while the camera recorded the laser beam position and enabled spatial point selection.

Supplementary Note 2. Theoretical model of cavity waveguide systems

Single-cavity waveguide system

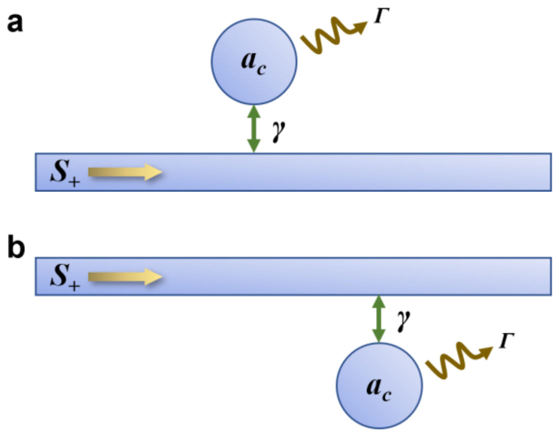

Supplementary Figure 2. Theoretical model of a single-cavity waveguide coupling system. The cavity is (**a**) above or (**b**) below the waveguide.

Dual-cavity waveguide system

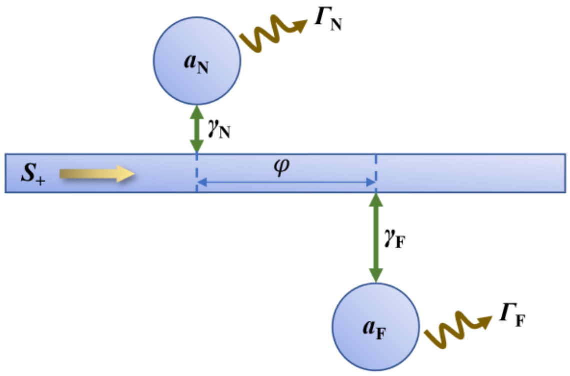

Supplementary Figure 3. Theoretical model of the dual-cavity waveguide coupling system. Subscripts N and F denote the near- and far-field cavities, respectively. The former lies above the waveguide whereas the latter lies below the waveguide.

Supplementary Note 3. Topological phases of the unit cell, and interface/cavity dispersions

Phononic crystals with $\theta = 20°$ and $\theta = -20°$ are designated PnC-A and PnC-B respectively. Supplementary Fig. 4 presents Berry curvatures for Sample 1, showing negative/positive concentrations near K/K' points (Supplementary Fig. 4a, top/bottom panels). Integration around K/K' yields valley Chern numbers [1, 2, 3], with $\theta = 20°$ exhibiting $C_v = \pm 0.42$ – closely matching theoretical $C_v = \pm 1/2$ values [1, 2, 3]. Valleys K_1 and K_2 exhibit opposing topological properties, further evidenced by opposite Berry curvatures for $\theta = \pm 20°$ at K_2 (Supplementary Fig. 4b), confirming topological contrast between PnC-A and B. Berry curvatures for Samples 2 and 3 are similar to Sample 1.

Supplementary Fig. 5a shows a supercell with PnC-A sandwiched between PnC-B layers, creating B-A and A-B interfaces. Projected band structures for Sample 1 (Supplementary Fig. 5b) reveal gapless topological edge state branches (bold lines) spanning 1.79-2.00 MHz within the band gap, whereas shaded regions denote bulk modes. Dashed blue lines mark B-A interface

states; solid red lines mark A-B interface states. Displacement u_z distributions at selected frequencies (point symbols) confirm interface confinement (Supplementary Fig. 5a). Samples 2 and 3 exhibit a downward-shifted topological edge state range (1.8-2.04 MHz, Supplementary Fig. 5c), with both samples maintaining ~0.2 MHz band gaps.

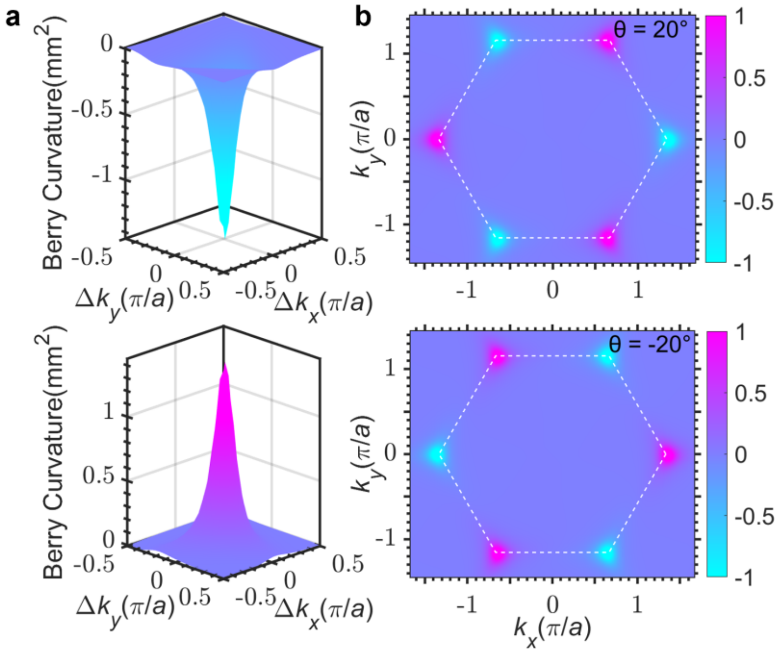

Supplementary Figure 4. Berry curvature of the valley PnC plate for Sample 1. a Berry curvature at valley K_2 (top panel) and K_1 (bottom panel) in **k**-space when $\theta = 20°$. **b** Berry curvature at K_2 when $\theta = \pm 20°$.

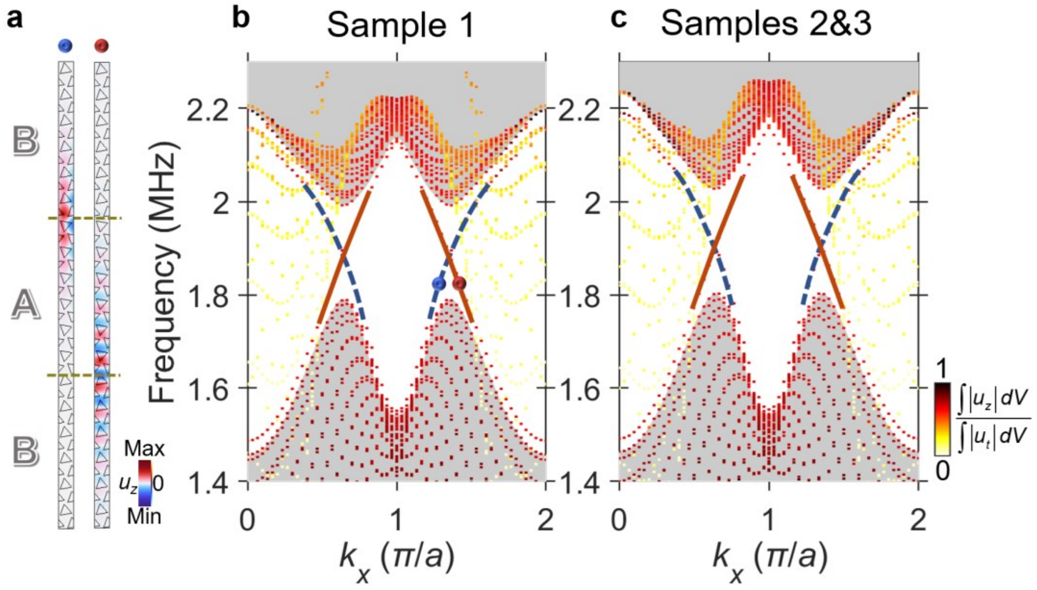

Supplementary Figure 5. Projected curves of the supercell B-A-B. a The supercell composed of two interfaces B-A and A-B. The TES is dominated by the component u_z. **b, c** Simulated dispersion for Sample 1 (s_l = 500 μm, h_p = 292 μm) in (**b**) and for Samples 2 and 3 (s_l = 526 μm, h_p = 289 μm) in (**c**). The color scale indicates the ratio of u_z to the total displacement u_t in the supercell.

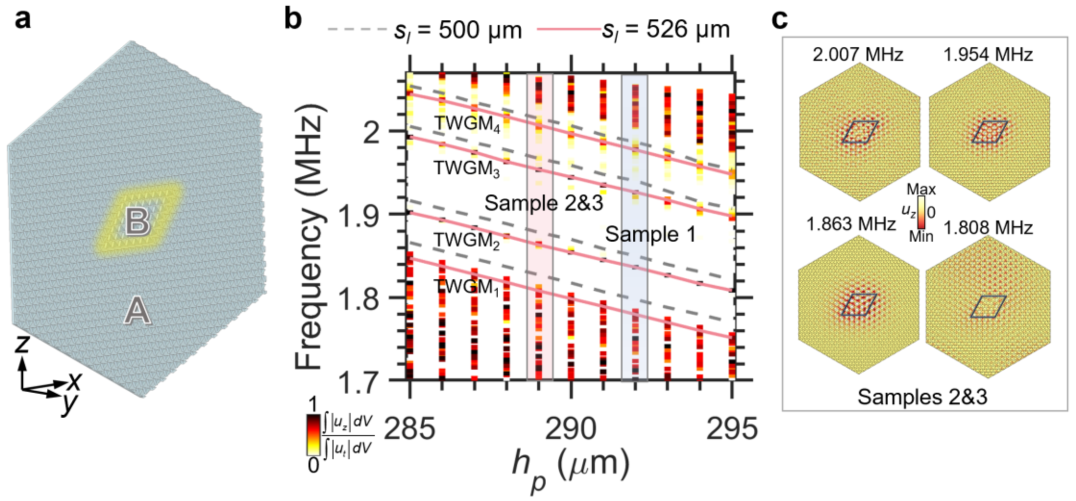

Supplementary Figure 6. Dispersion of cavity supercell. a The supercell composed of a rhombus-shaped phonon cavity. **b** Evolution of the eigenfrequencies of the supercell vs pillar height h_p, for pillar slide length s_l = 526 μm (pink solid lines) and 500 μm (gray dotted lines). The color scale indicates the ratio of u_z to the total displacement u_t in the supercell. **c** Distribution of u_z for TWGMs in Samples 2 and 3 (s_l = 526 μm, h_p = 289 μm).

We calculate the eigenfrequencies of the rhombus-shaped phononic cavity shown in Supplementary Fig. 6a. The hexagonal supercell contains a cavity with side length 7*a*, formed by inserting PnC-B into PnC-A. In Supplementary Fig. 6b, at s_l = 526 μm, the four pink lines correspond to TWGMs. The frequency of each TWGM decreases with increasing h_p. The pink box highlights TWGMs for Samples 2 and 3 at 1.808, 1.863, 1.954 and 2.007 MHz, respectively. Supplementary Fig. 6c shows their displacement distributions: at 1.863, 1.954 and 2.007 MHz, displacements are confined to the rhombus-shaped boundary, whereas at 1.808 MHz, the cavity displacement exhibits a bulk-mode distribution in the Pure PnC. The black dashed lines in Supplementary Fig. 6b represent results for s_l = 500 μm, where the TWGM frequencies are slightly higher than those shown by the pink solid lines. The four TWGMs of Sample 1 (denoted by the blue box) can be visualized from their field distributions in Fig. 2b. Notably, when the supercell configuration is reversed (inserting PnC-A inside PnC-B, opposite to Supplementary Fig. 6a), the TWGM eigenfrequencies remain nearly unchanged.

Supplementary Note 4. Results for single far-field cavity below the waveguide

The influence of cavity loss

Here we present theoretical and numerical results for the far-field coupling in the single cavity-waveguide system, referring to Figs. 2 and 3, for which case s_l = 500 μm, h_p = 292 μm. We perform theoretical fitting for the outlet $|u_z|$ corresponding to D_1-D_4 (in the left panels of Figs. 2c and 2d). We thereby obtain the dissipative and radiative losses of the cavity as a function of the distance D. As shown in Supplementary Fig. 7a, Γ and γ denote the dissipative and radiative losses of the cavity bridged with a Dirac strip, whereas Γ_g and γ_g refer to the cavity without a Dirac strip.

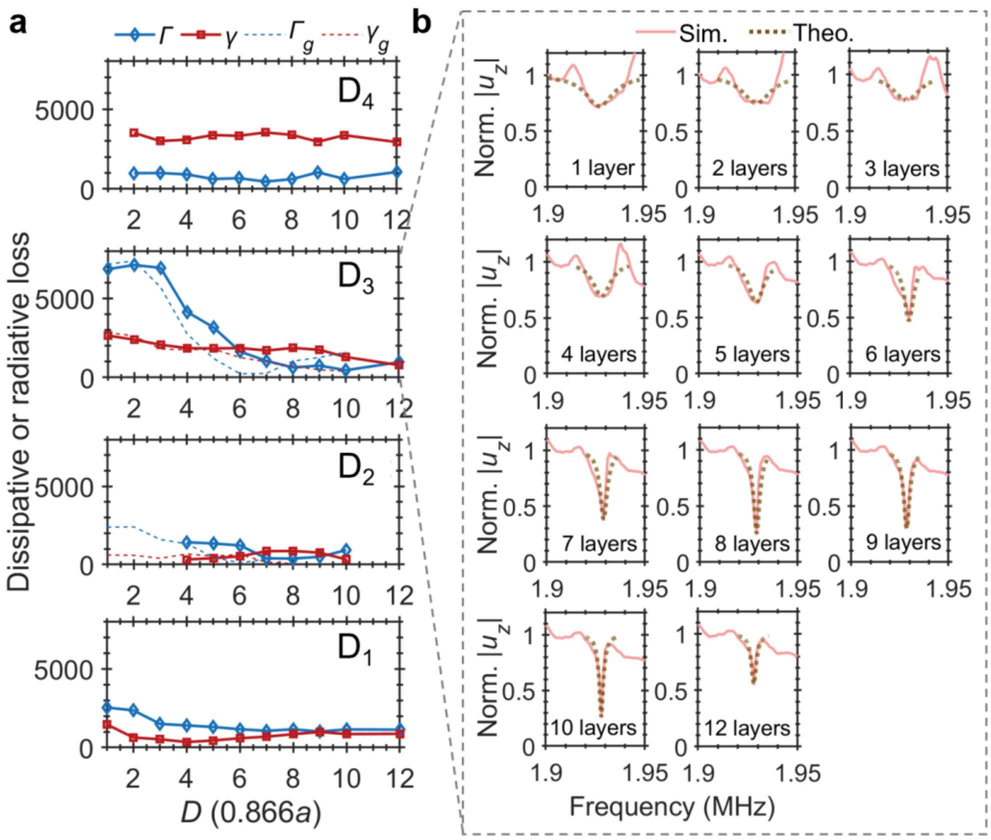

Supplementary Figure 7. Results of theoretical fitting for the single cavity-waveguide system in Fig. 1. a The dissipative and radiative losses of the cavity, with (solid lines) and without (dotted lines) a Dirac strip. **b** Normalized outlet $|u_z|$ for dip D_3 versus distance D.

In Supplementary Fig. 7a, we only present Γ and γ for D_1, since no strong dip occurs near 1.8 MHz for the case without a Dirac strip (left panel of Fig. 2d). Γ is larger than γ within D = 1–12 layers, except at D = 9 layers, without a significant outlet dip near 1.8 MHz (left panel of Fig. 2c). For D_2 in Supplementary Fig. 7a, $\Gamma = \gamma$ occurs at D = 7 and 9 layers when with the Dirac strip (solid line), leading to critical coupling and a transmission dark bar near 1.84 MHz (left panel of Fig. 2c), even in the far-field. In comparison, without the Dirac strip (dotted line), the crossover point between Γ_g and γ_g occurs near D = 5 and 7 layers. Thus, the Dirac strip alters the range of D where $\Gamma = \gamma$ and enhances far-field coupling at D_2. Notably, Γ is not constant in this work owing to additional eigenmodes of the Dirac strip and structural factors (e.g., the cavity approaches the truncated boundary as D increases).

For D_3 in Supplementary Fig. 7a, the Dirac strip shifts the range $\Gamma < \gamma$ from D = 4.5–7.5 layers (dashed line) to D = 6–12 layers (solid line), corresponding to the dip near 1.93 MHz in the left-hand panel of Fig. 2c. Therefore, the Dirac strip modifies the range of D where $\Gamma < \gamma$. At high frequencies (D_4 in Supplementary Fig. 7a), $\Gamma < \gamma$ holds for all D = 1–12 layers in the presence of the Dirac strip. Γ_g and γ_g are omitted for D_4, as no strong dip occurs without the Dirac strip (left-hand panel of Fig. 2d).

Using D_3 as an example, Supplementary Fig. 7b shows theoretical fitting results (dashed lines) at different D, consistent with numerical curves (red lines). The outlet $|u_z|$ dip deepens as D increases, reaching a minimum at D = 8 layers, then shallows with further increases in D.

The influence of pillar rotation of the Dirac strip

Returning to the FFC circuit in Fig. 2a, we examine the effect of pillar rotation in the Dirac strip (Supplementary Fig. 8a). As shown in Supplementary Fig. 8a (upper panel), we vary the rotation angle θ of pillars in the PnC-C strip (yellow rectangle) from 0° to 20°. The PnC-C constitutes the Dirac strip at $\theta = 0°$. For different θ, we extract the normalized $|u_z|$ at the outlet of the waveguide and the cavity's lower edge, identifying $|u_z|$ dips D_1–D_4 at the outlet and peaks P_1–P_4 at the cavity edge (Supplementary Fig. 8a).

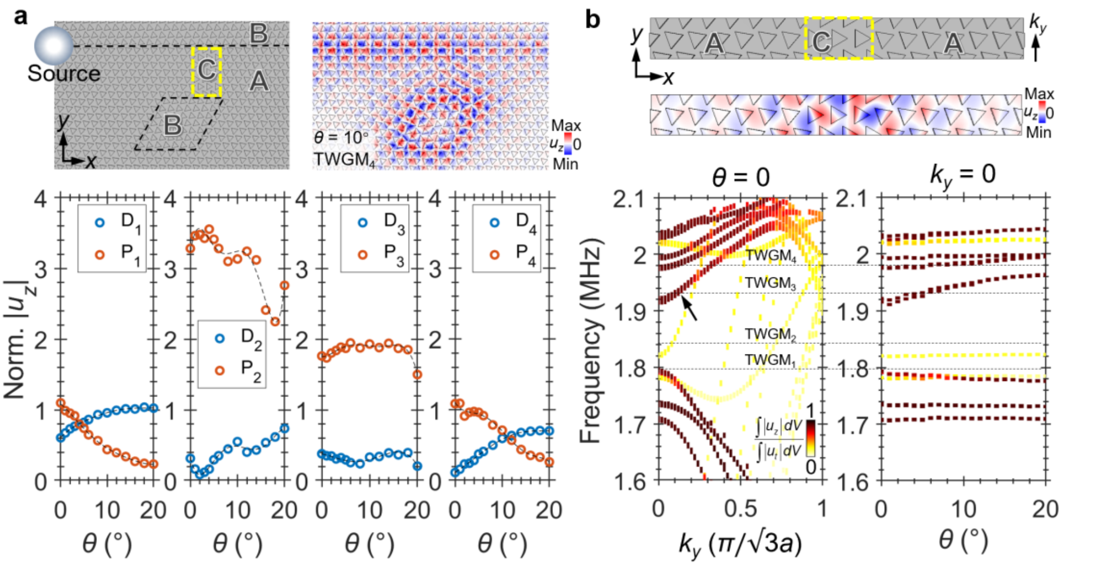

Supplementary Figure 8. The influence of pillar rotation in the PnC-C strip (based on the sample in Fig. 2a). a The FFC-waveguide structure and the u_z distribution when $\theta = 10°$ for TWGM4 (upper panel). Calculated normalized $|u_z|$ retrieved at the outlet (dip value, orange circle) and at the lower edge of the cavity (peak value, blue circle), versus the pillar rotation of the PnC-C in the model (upper panel). Dashed lines are fitted curves. PnC-C bridges the waveguide and the cavity, and becomes the Dirac strip when $\theta = 0$. **b** Supercell (upper panel) used to calculate the dispersion (lower left panel) when $\theta = 0^{\circ}$. The color scale indicates the ratio of u_z to the total displacement u_t in the supercell. The middle panel shows a map of u_z at 1.93 MHz when $\theta = 0^{\circ}$. Evolution of the eigenfrequencies (lower right panel) versus the pillar rotation for PnC-C when $k_y = 0$.

In the subgraph for D_1 and P_1, $|u_z|$ at D_1 increases gradually whereas $|u_z|$ at P_1 decreases gradually. The two profiles intersect at approximately $\theta = 4°$. Near $\theta = 10°$, $|u_z|$ at D_1 plateaus. For D_2 and P_2, $|u_z|$ at D_2 exhibits a sharp decrease from 0° to 2°, followed by a gradual increase from 2° to 20° with a minor peak around 10°. The value of $|u_z|$ at P_2 shows the opposite trend compared to the D_2 profile between 2° and 18°. D_3 and P_3 peak and dip values both remain stable from $\theta = 0°$ to 18°. However, $|u_z|$ at D_3 decreases gradually from 0° to 8°, then increases gradually from 8° to 18°. D_4 and P_4 pair exhibit a trend similar to D_1 and P_1, with the profiles crossing near $\theta = 10°$. At approximately $\theta = 10°$, $|u_z|$ at D_4 plateaus.

To provide further detail, a supercell was constructed, as depicted in the upper panel of Supplementary Fig. 8b. This supercell represents a portion extracted between the waveguide and cavity containing the Dirac strip. Periodic boundary conditions are applied along the y-direction, whereas continuity conditions are applied along the x-direction. The lower left panel of Supplementary Fig. 8b presents the dispersion for the supercell A-C-A structure at $\theta = 0°$ for

PnC-C. Thanks to the overlap between the bulk branch of the Dirac strip and the resonant frequency of the cavity for $TWGM_3$, highly-efficient matched coupling becomes possible between the horizontal waveguide and the far-field cavity. The lower right panel shows the evolution of this supercell's dispersion versus θ for PnC-C at $k_y = 0$. The middle panel illustrates a typical u_z field map for an antisymmetric plate wave at 1.931 MHz and $k_y = 0.12\pi/\sqrt{3}a$ when $\theta = 0°$. The u_z field is confined within the PnC-C, indicating that the PnC-C acts as a bridge guiding energy between the straight waveguide and the cavity.

In the lower left panel of Supplementary Fig. 8b, $TWGM_2$ lies within the bandgap for antisymmetric plate waves, whereas the other three TWGM modes overlap with branches of bulk modes. In the lower right panel, as θ increases to approximately 10°, it can be observed that $TWMG_1$ through $TWMG_3$ all fall within the bandgap for antisymmetric plate waves, whereas $TWGM_4$ intersects several bulk branches of antisymmetric plate waves. This explains why the $|u_z|$ profiles in Supplementary Fig. 8a exhibit different rates of variation or trends near the crossover angle of 10°. Nevertheless, the bulk branches of antisymmetric plate waves demonstrate a potential mechanism for PnC-C enhancement of the interaction between the straight waveguide and the cavity. However, evanescent waves may still contribute [4]. For instance, although $TWGM_2$ is within the bandgap, pillar rotation within PnC-C still noticeably affects the $|u_z|$ profile for D_2. In the subsequent models, we focus on the Dirac strip, and do not further consider the influence of pillar rotation in PnC-C.

The influence of material loss

The effects of material loss can be incorporated into the numerical simulations. Assuming a material loss factor $\alpha = 0.0025$, Supplementary Fig. 9a displays the profiles of normalized $|u_z|$ at the Pure PnC, FFC, and Outlet locations. In Supplementary Fig. 9a, the frequencies corresponding to the TWGMs (blue line) and the dips (red line) align with those shown in Fig. 3c. Dip D_3 remains distinct; conversely, D_1, D_2 and D_4 all become larger owing to the introduction of material losses. Notably, D_1 even exceeds P_1.

The u_z field depicted in Supplementary Fig. 9b reveals that some elastic waves propagate to the outlet region of the straight waveguide at frequencies corresponding to D_1, D_2 and D_4, but not at D_3. For reference, Supplementary Fig. 9c presents results without material losses, derived from Fig. 3c. Inside the cavity, a counter-clockwise energy flow (indicated by black arrows) is visible at D_1 through D_4, determining the overall flow direction marked by the green arrows in Fig. 3d.

Finally, the experimental u_z distribution at 1.808 MHz is included here (Supplementary Fig. 9d) as a supplement to Fig. 3b. $TWGM_1$ can be identified, although bulk waves are also present.

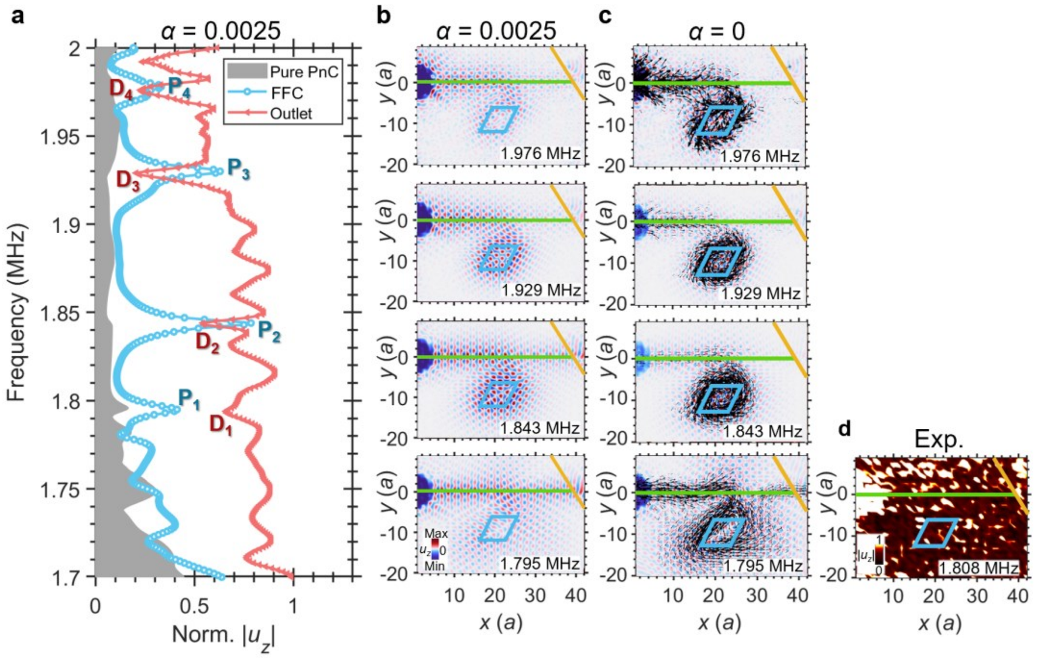

Supplementary Figure 9. The influence of material losses on an FFC-waveguide system (D = 7 layers). a, **b** Simulated normalized $|u_z|$ for the Pure PnC, FFC and Outlet (**a**), together with the u_z distribution (**b**), for the case in which the material loss factor $\alpha = 0.0025$. **c** The u_z distribution and energy flow for the case without material losses, at the same frequencies as in (**b**). **d** Measured $|u_z|$ distribution at 1.808 MHz.

Supplementary Note 5. Results for single cavity above the waveguide system

Another single cavity-waveguide circuit is presented in Supplementary Fig. 10a. In this configuration, a rhombus-shaped cavity is positioned above the waveguide, connected via the Dirac strip (shown in yellow). The figure shows results as a function of distance D for Sample 2 (s_l = 526 µm, h_p =289 µm). Supplementary Fig. 10b displays the computed map of normalized $|u_z|$ at the waveguide outlet and at the upper edge of the cavity. These simulations were performed with zero material loss.

There are four dark spots D_1-D_4, near 1.81, 1.865, 1.955 and 2.1 MHz, respectively (left-hand panel of Supplementary Fig. 10b). Compared to D_2-D_4, the dip D_1 is relatively shallow. Regarding D_2, the dip range is approximately D = 5 – 9 layers. The dip D_3 extends to D = 3 – 10 layers. The dip D_4 exists over the entire D = 1 – 12 layers. In right-hand panel of Supplementary Fig. 10b, the four light bars P_1-P_4 represent the TWGMs corresponding to D_1-D_4. The weak $TWGM_1$ near 1.81MHz causes the shallow dip D_1, whereas the relatively stronger $TWGM_2$-$TWGM_4$ cause the deeper dips D_2-D_4.

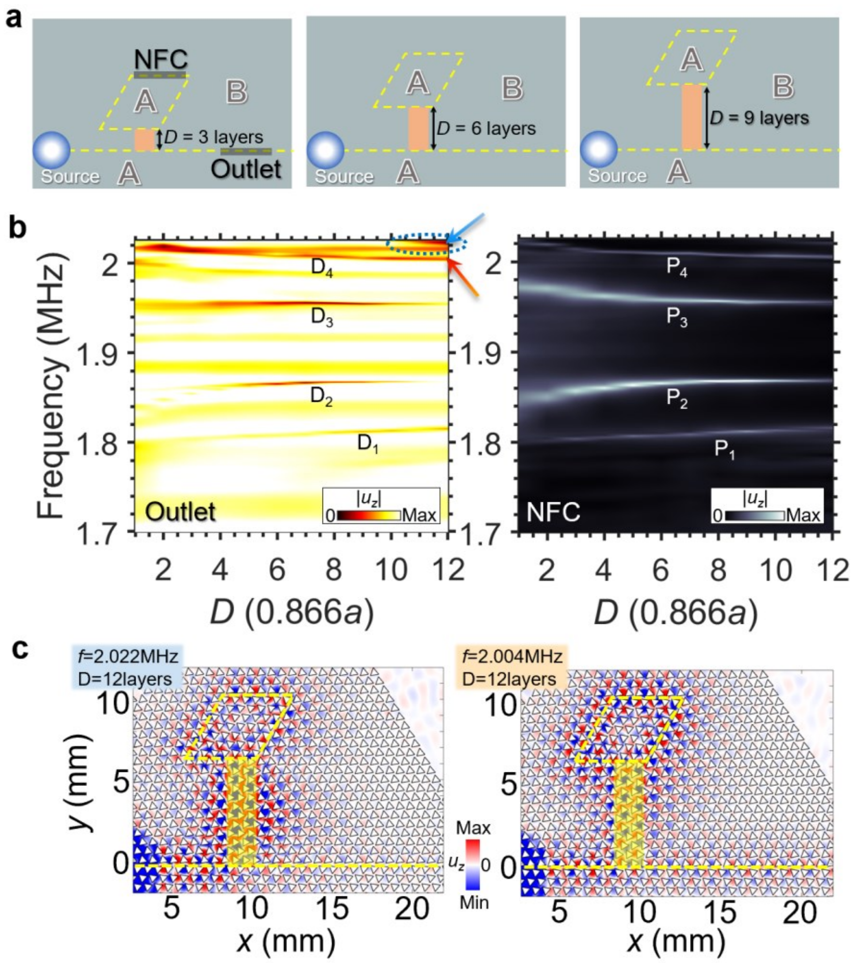

Supplementary Figure 10. Simulated results for a single cavity above a waveguide (Sample 2). a Diagram of one cavity above a waveguide for increasing values of *D*. **b** Left- and right-hand panels: normalized $|u_z|$ at the outlet and at the top edge of the cavity in (**a**), respectively, versus Dirac strip length *D*. **c** Left- and right-hand panels: the displacement field at 2.022 and 2.004 MHz, respectively, when *D* = 12 layers, denoted by the blue and red arrows in (**b**).

In Supplementary Fig. 10b, there are other dips besides those caused by TWGMs. For example, as *D* is quite large, the Dirac strip itself can possess resonant modes. Supplementary Fig. 10c shows the displacement fields when waveguide-Dirac strip coupling (left-hand panel) and waveguide-cavity coupling (right-hand panel) occur. Owing to the waveguide-Dirac strip coupling, there exists an additional transmission dip (blue dotted ellipse in Supplementary Fig. 10b). Since this work makes use of Dirac strips to enhance the coupling between a cavity and a waveguide, we avoid this phenomenon in our designs. The width of our Dirac strip contains only 3 columns of pillars, resulting in several advantages: *D* is large enough to be regarded as producing a far-field scenario; the coupling between the cavity and the waveguide is strong enough; the perimeter of the Dirac strip is not long enough to produce resonant modes therein.

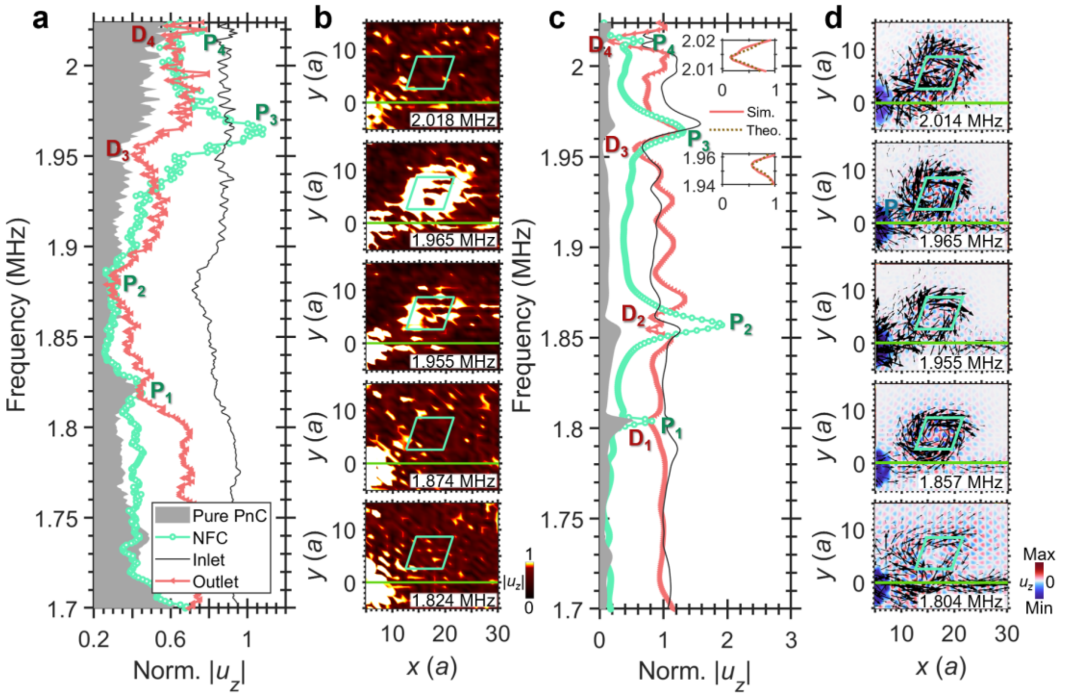

Supplementary Figure 11. Observation of enhanced coupling in a near-field cavity-waveguide system. a Measured normalized $|u_z|$ excited by a circular source upon generation of electrical pulses of central frequency f_c = 1.8 MHz. **b** The $|u_z|$ distribution at typical frequencies. **c** Simulated results corresponding to (**a**). The four TWGMs (blue line) match the four dips (red line) at 1.804, 1.857, 1.965 and 2.014 MHz. **d** The u_z distribution together with the in-plane energy flux (black arrows).

We now turn to the experimental study of a near-field cavity-waveguide sample, as shown in the left panel of Supplementary Fig. 10a. The cavity is positioned above the waveguide, bridged by Dirac strips with $D = 3 \times 0.866a$. Using established experimental and simulation techniques, we obtained profiles of $|u_z|$ at the Pure PnC (shaded region), NFC (green line), waveguide Inlet (black line), and Outlet (red line), as shown in Supplementary Figs. 11a and 11c, respectively. From the numerical results in Supplementary Fig. 11c, four peaks (P_1–P_4) appear in the NFC region, corresponding to four TWGMs. Additionally, two deep dips (D_3 and D_4) are observed.

The inset of Supplementary Fig. 11c shows numerical (solid lines) and theoretical (dashed lines) profiles of $|u_z|$ at D_3 and D_4. Experimentally, three minor peaks (P_1, P_2 and P_4) and one strong peak (P_3) are observed, along with one deep dip (D_3). These experimental results show fairly good agreement with numerical simulations.

For further detail, Supplementary Figs. 11b and 11d show mappings of $|u_z|$ at frequencies corresponding to P_1, P_2, D_3, P_3 and P_4, ordered from low to high frequency. Experimentally, the $|u_z|$ maps at P_1, P_2 and P_4 reveal perturbation by bulk waves within the Pure PnC region. Even numerically, the u_z maps indicate the presence of bulk waves in the Pure PnC. This explains why P_1, P_2, and P_4 exhibit relatively weak signatures in experiment.

Conversely, at D_3 in Supplementary Fig. 11a, almost no energy reaches the outlet, indicating enhanced coupling between the cavity and the straight waveguide. A frequency crossover also exists between D_3 (1.955 MHz) and P_3 (1.965 MHz), aligning well with numerical predictions. Furthermore, Supplementary Fig. 11d shows energy flow (black arrows) emanating from the left inlet along the straight waveguide and coupling into the cavity via the

Dirac strip. This implies a clockwise flow along the cavity path. At D_3, no wave propagates from the cavity toward the right-hand section of the waveguide.

Supplementary Note 6. Results for the dual-cavity waveguide system

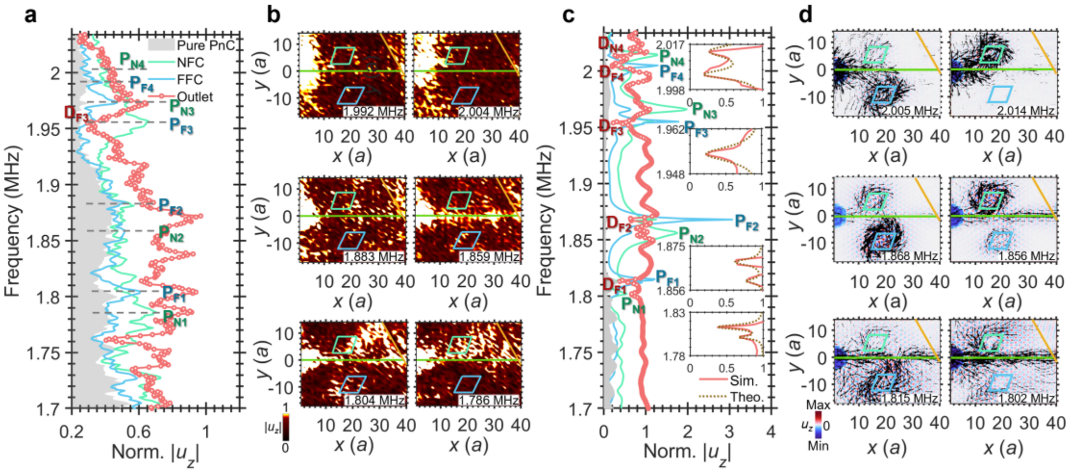

Supplementary Figure 12. Observation of enhanced coupling in the dual-cavity waveguide system. **a**, **b** Measured normalized $|u_z|$ at Pure PnC, NFC, FFC and Outlet in the dual-cavity system of Fig. 4a, together with the $|u_z|$ distribution (**b**). **c** Simulated results corresponding to (**a**). **d** The simulated u_z distributions together with the associated in-plane energy flux (black arrows).

Regarding the dual-cavity waveguide system, Supplementary Figs. 12a and 12c show profiles of normalized $|u_z|$ for the Pure PnC (gray region), NFC (green line), FFC (blue line), and Outlet (red line). From the experimental result in Supplementary Fig. 12a, it is somewhat difficult to directly distinguish the peaks in NFC/FFC or the dips in Outlet $|u_z|$ – which indicate TWGM locations and enhanced coupling – except for P_{N3}, P_{F3} and D_{F3}. We therefore label P_{N1}-P_{N4}, P_{F1}-P_{F1}, and D_{F1}-D_{F4} in Supplementary Fig. 12c, and subsequently mark P_{N1}-P_{N4} and P_{F1}-P_{F4} in Supplementary Fig. 12a.

Supplementary Fig. 12b presents the experimental $|u_z|$ maps at P_{F1}, P_{F2}, and P_{F4} (left-hand column panels) and at P_{N1}, P_{N2} and P_{N4} (right-hand column panels). Numerical u_z maps are shown in Supplementary Fig. 12d, including in-plane energy flux (black arrows). Experimentally, at P_{N1}, P_{N2} and P_{N4}, wave fields are confined to NFC with relatively weak $|u_z|$ in the FFC. Bulk waves are observed within the Pure PnC to the right of the cavities at P_{N1} and P_{N2}, and at the truncated PnC-silicon plate interface at P_{N4}. Conversely, at P_{F1}, P_{F2} and P_{F4}, wave fields are confined to both the NFC and FFC alongside bulk waves, with pronounced bulk waves at the truncated outlet interface for P_{F4}. These results demonstrate mutual excitation of the cavities by $TWGM_{N1}$, $TWGM_{N2}$, $TWGM_{N4}$ or $TWGM_{F1}$, $TWGM_{F2}$, $TWGM_{F4}$.

The outlet $|u_z|$ distribution is perturbed by bulk waves within the Pure PnC or along the truncated outlet interface, preventing clear observation of transmission dips. Note that the experimental setup makes use of 7-cycle sinusoidal pulses as a wave source to enhance the SNR (Signal-to-Noise Ratio). This source type permits signal reverberation to some extent, and explains the observed $|u_z|$ along the truncated outlet interface.

In Supplementary Fig. 12d, numerical results for u_z show phenomena similar to the experimental observations in Supplementary Fig. 12b at P_{N1}, P_{N2} and P_{N4}. However, u_z along

the truncated outlet interface is minimal in the simulation. The primary reason is the use of PML (Perfectly Matched Layers) to suppress boundary reflections at the silicon plate termination, an ideal condition not replicated experimentally. Simulations confirm wave confinement to the NFC and FFC at P_{N1}, P_{N2}, P_{N4} or P_{F1}, P_{F2}, P_{F4}, indicating mutual cavity excitation by $TWGM_{N1}$, $TWGM_{N2}$, $TWGM_{N4}$ or $TWGM_{F1}$, $TWGM_{F2}$, $TWGM_{F4}$. FFC exhibits larger u_z than NFC, and bulk waves are present at P_{F1} and P_{F2}.

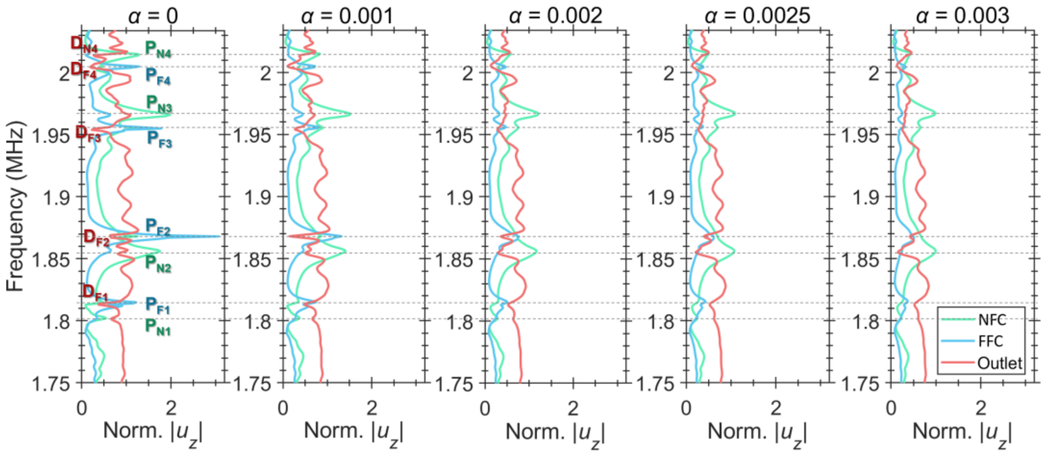

Supplementary Figure 13. Numerical calculation of the influence of material losses on the dual-cavity waveguide system, for loss factors α = 0-0.003. The loss factor increases sequentially from left to right, with an interval of 0.001. The green, blue, and red lines shows the normalized $|u_z|$ at NFC, FFC, and Outlet respectively. Dotted lines present peaks P_{N1}-P_{N4} and P_{F1}-P_{F4}.

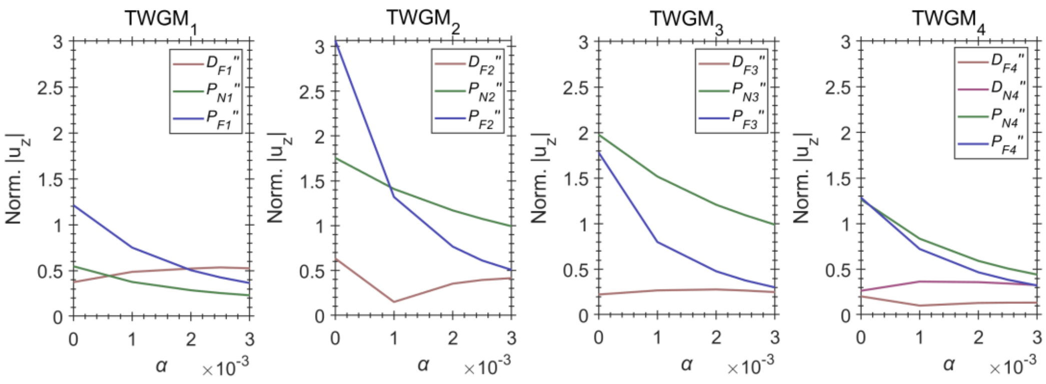

Supplementary Figure 14. Calculated profiles of normalized $|u_z|$ at the frequencies of D_{F1}-D_{F4}, P_{N1}-P_{N4} and P_{F1}-P_{F4}, respectively, caused by TWGMs in the FFC for the dual-cavity system. Only D_{N4} is shown to be caused by $TWGM_4$ in the NFC in dual-cavity system. All these results are retrieved from Supplementary Fig. 13.

To investigate the influence of material loss, the material loss factor α is introduced in the dual-cavity circuit simulation. Supplementary Fig. 13 shows the profiles of normalized $|u_z|$ for the NFC (green line), the FFC (blue line), and the Outlet (red line). The left panel of Supplementary Fig. 13 shows the results without material loss, identical to Supplementary Fig. 12c. The fine dashed lines in Supplementary Fig. 13 label the frequencies P_{F1}-P_{F4} and D_{F1}-D_{F4} caused by the FFC, along with the frequencies P_{N1}-P_{N4} and D_{F4} related to the NFC. One can see that these frequencies remain almost unchanged even as material loss increases.

For detailed comparison, Supplementary Fig. 14 summarizes the evolution of the

calculated peaks and dips: (P_{F1}, P_{N1}, D_{F1}), (P_{F2}, P_{N2}, D_{F2}), (P_{F3}, P_{N3}, D_{F3}) and (P_{F4}, P_{N4}, D_{F4}, D_{N4}). As α increases, the $|u_z|$ peaks P_{N1}-P_{N4} and P_{F1}-P_{F4} decrease rapidly.

The outlet $|u_z|$ dip evolution is as follows: for dip D_{F1}, the outlet $|u_z|$ gradually increases from 0.37 ($\alpha = 0$) to 0.52 ($\alpha > 0.001$). Dip D_{F2} features a V shape, increasing sharply from $\alpha = 0.001$ to $\alpha = 0.002$, meaning certain material loss can minimize its value. For D_{F3}, the outlet $|u_z|$ value gradually increases from 0.22 ($\alpha = 0$) to 0.27 ($\alpha > 0.001$). Above 2 MHz, dip D_{F4} maintains a small value around 0.15 but also features a V shape. Dip D_{N4} increases quickly, causing critical coupling to disappear when $\alpha > 0.001$.

In brief, material loss rapidly reduces the cavity $|u_z|$ peaks. However, the outlet $|u_z|$ dips, such as D_{F2} and D_{F4}, can reach their smallest values as the material loss factor increases. This provides useful clues for attaining exceptional points in future phonon systems.

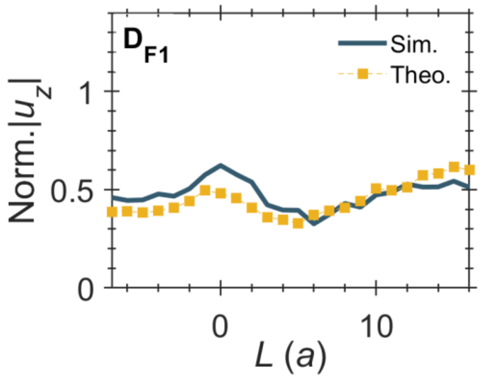

Supplementary Figure 15. Distance-dependent evolution of normalized $|u_z|$ extracted from D_{F1} near 1.815 MHz, together with the corresponding theoretical result. The simulated and theoretical results are shown by the solid line and squares respectively.

Supplementary Fig. 15 shows the profiles of $|u_z|$ versus cavity distance L in the dual-cavity waveguide system corresponding to the low-frequency dip D_{F1} in Fig. 5a. The solid line represents the results extracted from Fig. 5a, whereas the orange line denotes the theoretical prediction. The simulated and theoretical results exhibit good agreement across the entire range of L.

Supplementary References

1. Wang, J. & Mei, J. Topological valley-chiral edge states of Lamb waves in elastic thin plates. *Appl. Phys. Express* **11**, 057302 (2018).
2. Wu, X. *et al.* Direct observation of valley-polarized topological edge states in designer surface plasmon crystals. *Nat. Commun.* **8**, 1304 (2017).
3. Lu, J. *et al.* Observation of topological valley transport of sound in sonic crystals. *Nat. Phys.* **13**, 369-374 (2016).
4. Hatanaka, D. *et al.* Valley pseudospin polarized evanescent coupling between microwave ring resonator and waveguide in phononic topological insulators. *Nano Lett.* **24**, 5570-5577 (2024).
