# Supplementary material for: Far-field phonon coupling in valley metamaterial circuits
Source: Nat Commun. 2025 Dec 11;17:422. doi: 10.1038/s41467-025-67108-6 (PMC12796208; doi:10.1038/s41467-025-67108-6)
Supplement: Supplementary file 2 — Description of Additional Supplementary Files [file 41467_2025_67108_MOESM2_ESM.pdf]

Description of Supplementary Files

File Name: Supplementary Movie 1

Description: A video demonstration of the spatiotemporal displacement u_z field in the single far-field waveguide-cavity phonon system at 1.975 MHz. Lines outline the waveguide, cavity path, and truncated terminal.

File Name: Supplementary Movie 2

Description: A video demonstration of the spatiotemporal displacement u_z field in the dual-cavity waveguide phonon system at 1.955 MHz. Lines outline the waveguide, cavity path, and truncated terminal.
