# Supplementary material for: Author Correction: Far-field phonon coupling in valley metamaterial circuits
Source: Nat Commun. 2026 Apr 27;17:3825. doi: 10.1038/s41467-026-71465-1 (PMC13121707; doi:10.1038/s41467-026-71465-1)
Supplement: Supplementary file 1 — Original, uncorrected article [file 41467_2026_71465_MOESM1_ESM.pdf]

Original, uncorrected article

nature communications

Article

https://doi.org/10.1038/s41467-025-67108-6

Far-field phonon coupling in valley metamaterial circuits

Received: 16 July 2025

Accepted: 21 November 2025

Published online: 11 December 2025

Check for updates

Yao Huang[1], Weitao Yuan[2], Zhiwei Guo[3], Qi Wang[4], Yuxuan Zhang[1], Yueting Zhou[1], Yongdong Pan[1], Jia Zhou[5], Oliver B. Wright[6], Zheng Zhong[7] & Jinfeng Zhao[1]

On-chip whispering-gallery-mode cavities enable versatile bosonic wave manipulation but typically rely on near-field evanescent coupling. Here we experimentally demonstrate broadband far-field phonon coupling in a valley metamaterial cavity integrated with a Dirac-cone waveguide—termed a "Dirac strip". The far-field coupling is confirmed by transmission spectroscopy and spatiotemporal field mapping over distances up to approximately five wavelengths, enabling multiplexed, distance-robust coupling pathways that overcome near-field limitations. By combining near-field and far-field cavities on the same substrate, we achieve amplification and control of non-Hermitian dynamics through loss and distance modulation of sympathetic resonances, directly resolved via piezo-laser interferometry. This work establishes a scalable phononic platform for far-field coupling and paves the way for parallel topological wave processors.

Whispering gallery modes (WGMs), characterized by continuous reflections along curved cavity interfaces[1], have become foundational elements in waveguide-cavity coupling systems with broad utility across acoustics[2], elastodynamics[3], optics[4], and electronics[5]. These modes enable key functionalities including low-threshold lasing[6], enhanced sensing[7], and critical coupling – where dissipative loss (Γ) equals radiative loss (γ)[8]. The pursuit of advanced on-chip coupling schemes has garnered considerable interest in scalable information processing[3–5,8].

Recent advances in enhancing waveguide-cavity coupling have exploited non-Hermitian physics in waveguide-cavity systems through deliberate modulation of external loss and gain. This approach enables phenomena such as exceptional points[9,10] that yield nonlinear dynamics[11], nonreciprocal transmission[12], and spectral flows[13,14]. Non-Hermitian systems are non-conservative and characterized by energy exchange[15,16], encompassing three situations: loss only, gain only, or both. Concurrently, topological metamaterials have transformed continuum waveguide and cavity design, giving rise to topological edge states (TESs)[17,18], topological whispering gallery modes (TWGMs)[19–21], and corner states[22,23]. These bosonic wave counterparts encode topological protection and synthesized chirality[24,25], liberating cavity designs from perfect circular geometries[26,27]. Valley-phononic TWGMs can exhibit Hermitian[20] or non-Hermitian behaviour through unit cell-level modulation of gain and loss[26].

Despite these advances, achieving far-field coupling remains a fundamental challenge in waveguide-cavity systems[28]. All approaches for coupling in such systems to date rely on evanescent near-fields, which intrinsically confine energy transfer to sub-wavelength distances. Far-field coupling schemes, by contrast, if achievable would enable unprecedented spatial flexibility for on-chip integration, full utilization of wave degrees of freedom, and direct observation of intercavity dynamics in multi-resonator architectures. Yet their construction has not been possible owing to the absence of broadband, robust, and distance-insensitive coupling mechanisms.

[1]School of Aerospace Engineering and Applied Mechanics, Tongji University, 100 Zhangwu Road, Shanghai 200092, China. [2]Sichuan Province Key Laboratory of Advanced Structural Materials Mechanical Behavior and Service Safety, School of Mechanics and Aerospace Engineering, Southwest Jiaotong University, Chengdu, Sichuan 611756, China. [3]MOE Key Laboratory of Advanced Micro-Structured Materials, School of Physics Science and Engineering, Tongji University, Shanghai 200092, China. [4]School of Microelectronics, Nanjing University of Science and Technology, Nanjing 210094, China. [5]State Key Laboratory of ASIC and System, School of Microelectronics, Fudan University, Shanghai 200433, China. [6]Hokkaido University, Sapporo, Hokkaido 060-0808, Japan. [7]School of Science, Harbin Institute of Technology, Shenzhen 518055, China. e-mail: jinfeng.zhao@tongji.edu.cn

In this work, we experimentally demonstrate broadband, robust waveguide-cavity far-field coupling. This is achieved through topological phonon interactions in on-chip valley waveguide-cavity systems using a Dirac-cone waveguide strip (a 'Dirac strip'), which enables long-distance coupling beyond conventional near-field limits and markedly improves coupling efficiency. This topological channel mediates enhanced waveguide-cavity coupling through the broadband and robust features of valley metamaterials. By co-locating near- and far-field cavities, we observe sympathetic resonances—simultaneous resonance in both cavities at a single frequency—and reveal non-Hermitian dynamics modulated by material loss and inter-cavity distance.

Results

Valley metamaterial circuits: three configurations

Three valley metamaterial circuits are investigated in this work (Fig. 1a). Sample 1 is a far-field cavity (FFC) waveguide circuit composed of triangular pillars, with side length $s_l = 500\,\mu m$ and height $h_p = 292\,\mu m$ (see Fig. 1b). Samples 2 and 3 correspond to the near-field cavity (NFC) waveguide circuit and dual-cavity waveguide circuit, respectively, with $s_l = 526\,\mu m$ and $h_p = 289\,\mu m$. All samples share the same lattice constant $a = 641\,\mu m$ and wafer thickness $e_t = 525\,\mu m$, with geometrical parameters detailed in Supplementary Table 1. The rhombus-shaped cavities have side length $7a$, and the waveguides span $38a$ horizontally. Rightward terminal boundaries are oblique to suppress reflections. The Dirac strips maintain a $3a$ width horizontally, extending vertically 7 layers for FFC structures and 3 layers for NFC structures. In dual-cavity configurations, the Dirac strips of NFC and FFC are positioned with a $7a$ (center-to-center) horizontal separation. Simplified models of the three samples are shown in Supplementary Figs. 2 and 3.

We fabricated valley phononic crystal (PnC) plates by etching arrays of triangular pillars in a honeycomb lattice (see Methods). Dirac cones appear at the K point of the Brillouin zone for $\theta = 0°$ (Fig. 1c, d, upper panels), while material anisotropy introduces a minor band gap (insets). Rotating the pillars to $\theta = 20°$ (phase A) and $\theta = -20°$ (phase B) generates broad band gaps for antisymmetric plate waves (Fig. 1c, d, lower panels), with slight frequency shifts of the Dirac cones between Sample 1 and Samples 2–3.

The unit cell of triangular pillars possesses C_{3v} symmetry when θ equals zero, and topological phase transitions are induced by changing the rotation angle θ to break this symmetry. These valley PnCs support the topological states reported previously[24,29]. We adopt this classic paradigm and fabricate all three samples on silicon wafers to explore novel mechanisms of wave propagation.

Single far-field waveguide-cavity phonon system

Our first on-chip waveguide-cavity phonon system integrates an FFC coupled to a straight waveguide via a Dirac strip (Fig. 2a), to enable the observation of far-field coupling. PnCs A and B exhibit opposite

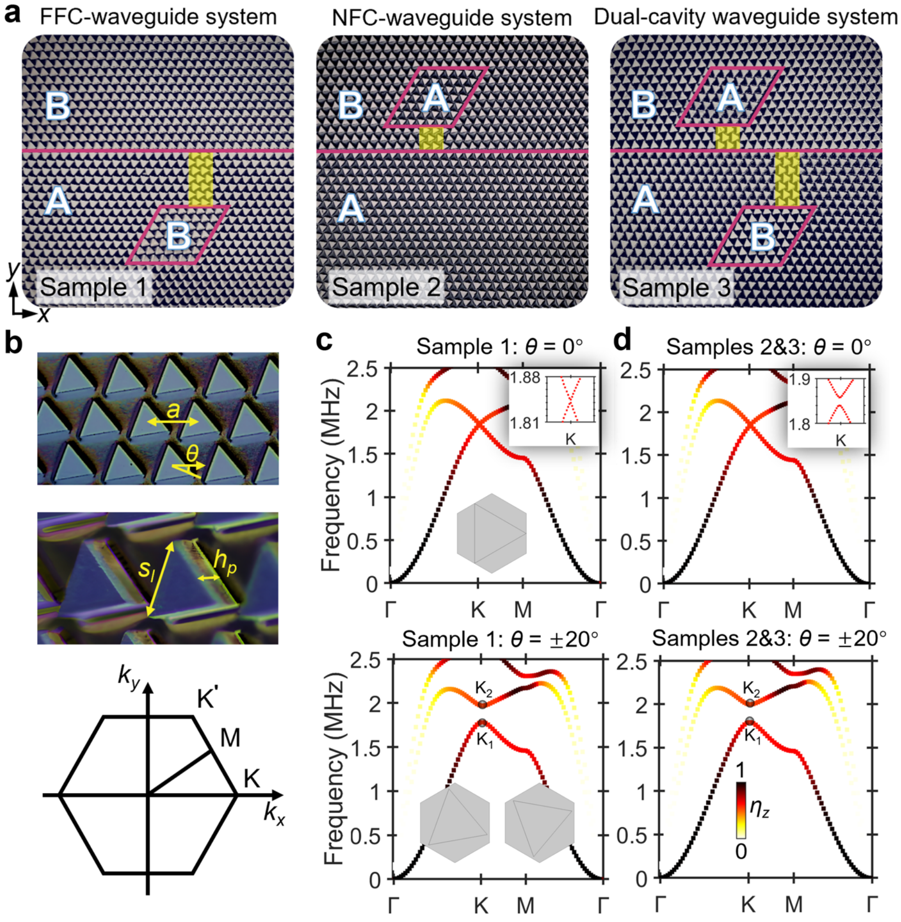

Fig. 1 | Samples and dispersion relations for the valley phononic plates. **a** Experimental samples for the (1) FFC-waveguide, (2) NFC-waveguide, and (3) dual-cavity waveguide circuits. The crystallographic directions [100] and [001] of silicon are aligned with the x- and z-axes, respectively. **b** (top) PnC plate, (middle) unit cell, and (bottom) Brillouin Zone taken from Sample 1. Geometrical parameters include lattice constant a, pillar side length s_l, pillar height h_p, silicon wafer thickness e_t, and substrate thickness $e_s = e_t - h_p$. Topological phases are controlled by tuning the rotation angles θ of the triangular pillars. **c**, **d** Band structures of the unit cell for $\theta = 0°$ (top panels) and $\theta = \pm 20°$ (bottom panels) are shown for Sample 1 in (**c**) and Samples 2 and 3 in (**d**). The color scale indicates the ratio of $|u_z|$ to the total displacement $|u_t|$ in the unit cell, defined by $\eta_z = \int |u_z| dV / \int |u_t| dV$. Insets show unit cell schemes and a zoom-in of the dispersion for $\theta = 0°$.

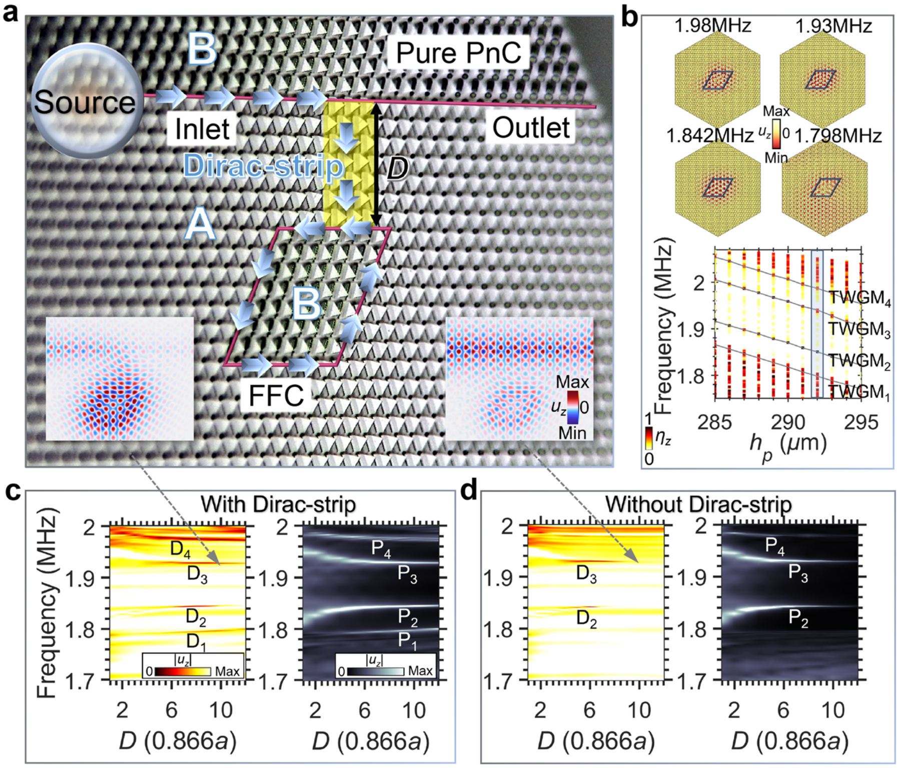

Fig. 2 | Far-field critical coupling in a Dirac-strip-enhanced waveguide-cavity phonon system. a Optical micrograph of the on-chip far-field cavity (FFC) connected to straight waveguide via a Dirac strip ($D = 7 \times 0.866a$, highlighted in yellow). Adjacent topologically distinct phononic crystals (PnCs) A and B define interfaces (red lines), along which blue arrows indicate the incident wave path. **b** Top: u_z distributions for $TWGM_1$-$TWGM_4$ when $h_p = 292$ μm. Bottom: TWGM eigenfrequencies as a function of the pillar height h_p with $s_l = 500$ μm. The colour map represents the ratio of $|u_z|$ to the total displacement $|u_t|$ in the supercell. **c** Computed map of normalized $|u_z|$ at the waveguide outlet (left) and cavity bottom edge (right), plotted against frequency and cavity-waveguide distance (i.e., the Dirac strip length) D, with the Dirac strip shown in (**a**). Left inset (shown in (**a**)): field distribution showing enhanced coupling at 1.93 MHz ($D = 10 \times 0.866a$). **d** As (**c**) but with a PnC-A domain instead of the Dirac strip. Right inset (shown in (**a**)): map of u_z at 1.93 MHz for $D = 10 \times 0.866a$, in the undercoupled state.

topological phases (Supplementary Fig. 4), and their interface supports topological edge states (TESs, Supplementary Fig. 5) and topological whispering gallery modes (TWGMs, Fig. 2b and Supplementary Fig. 6).

The cavity eigenfrequencies result from embedding PnC-B within a PnC-A domain (Fig. 2b). Within the antisymmetric plate wave band gap, four TWGMs ($TWGM_1$-$TWGM_4$) are identified (black solid lines), exhibiting a decrease in frequency with increasing h_p. For the experimental sample ($h_p = 292$ μm), the $TWGM_1$ at 1.798 MHz couples to bulk modes, evidenced by u_z field leakage beyond the cavity. In contrast, $TWGM_2$ (1.842 MHz), $TWGM_3$ (1.93 MHz) and $TWGM_4$ (1.98 MHz) remain confined within the band gap of both PnCs A and B, with u_z distributions localized along the cavity path.

To characterize the far-field phonon coupling, we show in Fig. 2c computed maps of normalized $|u_z|$ at the waveguide outlet (left panel) and cavity edge (right panel) plotted versus frequency and cavity-waveguide distance D, using a model matching the experimental sample in Fig. 2a. With the Dirac strip ($\theta = 0°$) bridging the cavity and waveguide, four outlet $|u_z|$ minima (D_1-D_4) emerge and shift toward the $TWGM_1$-$TWGM_4$ eigenfrequencies as D increases, whereas four cavity $|u_z|$ maxima (P_1-P_4) simultaneously develop, confirming TWGMs generation. These TWGMs enable enhanced coupling at large D—e.g., outlet $D_3 = 0.27$ at $D = 10 \times 0.866a$ and 1.93 MHz (see the left inset in Fig. 2a, corresponding to Fig. 2c). By comparison, Fig. 2d shows Dirac-strip-free counterparts—in which the Dirac strip is replaced by a PnC-A domain—where only minima D_2-D_3 persist (D_1/D_4 absent). These minima vanish rapidly with increasing D owing to diminished amplitude of $TWGM_2$-$TWGM_3$, yielding poor energy transfer (e.g., at $D = 10 \times 0.866a$ and 1.93 MHz, see the right inset in Fig. 2a, corresponding to Fig. 2d).

This enhanced far-field coupling depends on the cavity dissipation loss Γ, radiation loss γ, and resonance ω_0 (Supplementary Fig. 2). The transmission coefficient $T = \left|1 - \frac{\gamma}{i(\omega-\omega_0)+\Gamma+\gamma}\right|$ reaches a minimum value $T_{\min} = \frac{\Gamma}{\Gamma+\gamma}$. With the Dirac strip (Supplementary Fig. 7), the effect of the value of γ dominates over that of Γ even at large D, amplifying far-field coupling. This enhancement stems from the Dirac strip's topological protection and broadband properties[30,31]. The modeled antisymmetric bulk-wave branches emerge with wave energy confined to the Dirac strip, whereas their frequency ranges occupy the eigenfrequencies of $TWGM_1$, $TWGM_3$ and $TWGM_4$ (Supplementary Fig. 8). These guided bulk-wave branches are modulated by strip pillar rotation. For example, at strip pillar angle $\theta = 10°$, these bulk-wave branches occupy exclusively the $TWGM_4$ frequency, directly shaping the D_1-D_4 and P_1-P_4 $|u_z|$ versus θ profiles (Supplementary Fig. 8).

In situ time-resolved observations are performed by spatially mapping u_z over the waveguide-cavity circuit (see Methods). Normalized $|u_z|$ is measured at representative positions: Pure PnC (i.e., unmodified PnC), FFC, Inlet, and Outlet (Fig. 2a). As shown in Fig. 3a, the FFC $|u_z|$ (blue curve) exhibits peaks P_1-P_4 at 1.808, 1.867, 1.937 and 1.976 MHz, corresponding to the calculated frequencies of $TWGM_1$-$TWGM_4$. Compared to the Pure PnC case (gray background), the outlet $|u_z|$ (red curve) shows distinct minima at $D_3 \approx 0.32$ (1.937 MHz) and $D_4 \approx 0.30$ (1.976 MHz).

The $|u_z|$ field maps confirm incident wave coupling from waveguide to cavity (Fig. 3b). Minimal energy reaches the truncated outlet at frequencies corresponding to D_3/D_4 owing to $TWGM_3$/$TWGM_4$ excitation, whereas partial wave leakage at D_2 prevents outlet dip observation. The map of $|u_z|$ at D_1 is shown in Supplementary Fig. 9,

https://doi.org/10.1038/s41467-025-67108-6

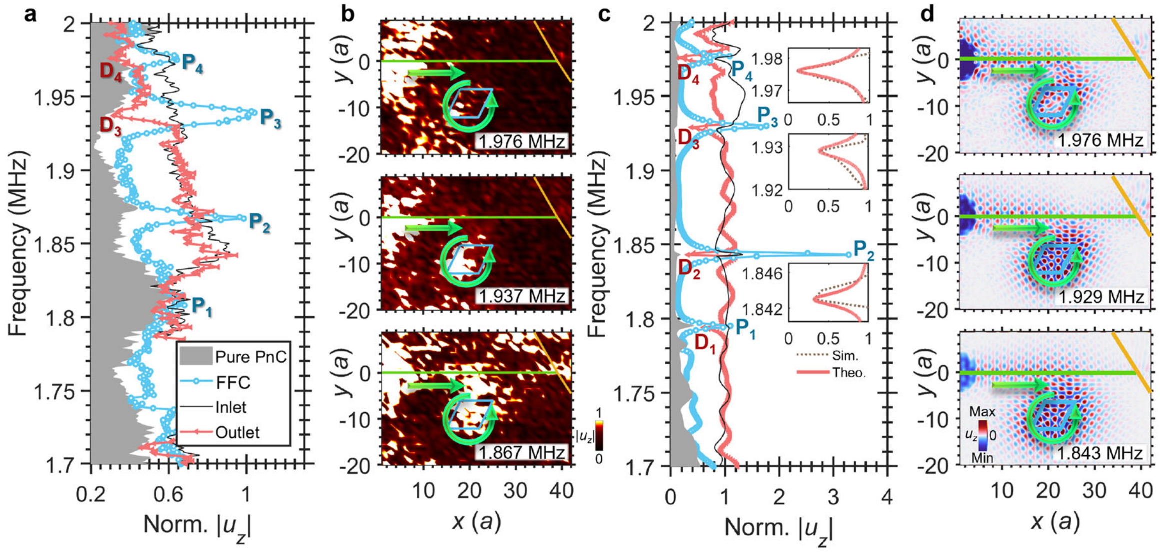

Fig. 3 | Enhanced coupling in a far-field cavity-waveguide phonon system. **a** Normalized $|u_z|$ measured at locations on the sample as marked in Fig. 2a (Pure PnC, FFC, Inlet, Outlet), excited by a pulsed source centered at $f_c = 1.8$ MHz. FFC spectrum (blue curve) shows peaks P_1-P_4 at 1.808, 1.867, 1.937 and 1.976 MHz, respectively. **b** Measured $|u_z|$ distributions at frequencies corresponding to P_2-P_4, together with the in-plane wave flux (bold arrows). Lines outline the waveguide, cavity path, and truncated terminal. **c** Simulated spectra showing FFC peaks P_1-P_4 (blue) and outlet dips D_1-D_4 (red) at 1.794, 1.843, 1.929 and 1.976 MHz. Insets show zoomed-in views of the outlet $|u_z|$ at D_2-D_4: numerical data are shown by the dotted lines, and theoretical results by the red solid lines. **d** Simulated u_z distributions at frequencies corresponding to P_2-P_4 with in-plane energy flux (bold arrows).

with generation of $TWGM_1$ and bulk waves in the Pure PnC. The time-resolved u_z map reveals anticlockwise circulation along the cavity path (Supplementary Movie S1).

Figure 3c, d show numerical counterparts to Fig. 3a, b. In Fig. 3c, FFC $|u_z|$ (blue curve) exhibits peaks P_1-P_4 consistent with experimental results (<0.024 MHz frequency shift). Outlet $|u_z|$ (red curve) shows dips D_1-D_4 from $TWGM_1$-$TWGM_4$ generation, visualized via u_z distributions in Fig. 3d. Distance $D = 3.9$–4.5λ (relative to TES wavelengths at frequencies corresponding to $TWGM_2$-$TWGM_4$) confirms a remarkable far-field coupling. The anticlockwise energy flow is revealed in Fig. 3d, matching experimental Supplementary Movie S1. The $TWGM_1$ generation at P_1/D_1 is perturbed by bulk waves in the Pure PnC, and the experimental perturbation by bulk waves at P_1/D_1 is verified, as shown in Supplementary Fig. 9.

Via dynamical equations (see Methods), Fig. 3c inset displays zoomed-in numerical (dashed) and theoretical (solid) outlet $|u_z|$ profiles at D_2-D_4. Radiative loss γ exceeds dissipative loss Γ (the overcoupled regime) for all TWGMs owing to Dirac-strip enhancement. Material loss ($\alpha = 0.0025$) moderately affects D_3/D_4 coupling (Supplementary Fig. 9) but significantly weakens D_1/D_2, whereas cavity peak frequencies (P_1-P_4) remain stable. Besides material loss and the influence of bulk waves, the experiment also encounters the effects of backward scattering[32] (arising from cavity corners[20]) and boundary effects at the metamaterial–silicon interface, whereas the input waves are limited in number and pulse power (see Methods). These factors can prevent waves from fully propagating along the ring path as in idealized simulations. While the full demonstration of wave fields relies on simulations, experiments remain essential to reveal the functional capabilities of the waveguide circuits, serving as a foundation for future applications. Far-field phonon coupling enables long-distance cavity energy localization and unlocks functionalities in complex systems, such as dual-cavity configurations.

Another near-field system demonstrates the near-field coupling. A single NFC-waveguide circuit ($D = 3\times0.866a$, Supplementary Fig. 10) produces dip D_3 from $TWGM_3$. Experimentally, we position the NFC above the Dirac-strip-bridged waveguide. Despite geometric variations compared to the FFC system, D_3/P_3 persist in experiment/simulation (Supplementary Fig. 11) regardless of cavity position. TWGM frequencies shift with change in s_l and h_p, but the robustness of D_3 confirms the existence of stable coupling. Crucially, NFC exhibits a 10 kHz frequency shift from D_3 (1.955 MHz) to P_3 (1.965 MHz) – unobserved in the FFC systems.

Dual-cavity waveguide system

Our final waveguide-cavity phonon system integrates both FFC and NFC, bridged to a shared waveguide via two Dirac strips (Fig. 4a). The cavities are positioned on opposite sides to prevent direct cavity-to-cavity coupling. With parameters matching the single NFC circuit parameters, we measure u_z to obtain $|u_z|$ at key positions (Fig. 4a). Focusing on $TWGM_3$ which generates transmission dips in individual cavities, the top panel of Fig. 4b shows peak values in $|u_z|$ of 0.66 (NFC) and 0.34 (FFC) at 1.956 MHz (the FFC $TWGM_3$ frequency), and corresponding values of 0.53 (NFC) and 0.47 (FFC) at 1.973 MHz (the NFC $TWGM_3$ frequency). This confirms sympathetic resonances[33] at each $TWGM_3$ frequency—i.e., both cavities are resonating simultaneously—validated by the $|u_z|$ field maps (middle/bottom panels in Fig. 4b). Wave energy is thus exchanged between cavities. The time-resolved u_z map (Supplementary Movie S2) reveals clockwise circulation in the upper cavity and anticlockwise circulation in the lower cavity.

In the top panel of Fig. 4b, the outlet $|u_z|$ shows a dip D_{F3} at 1.956 MHz. Isolated FFC measurements confirm that the D_{F3} dip originates from the resonance of the $TWGM_3$ in the FFC, whereas resonance hybridization in the dual-cavity system additionally couples D_{F3} to the $TWGM_3$ in the NFC. This overlap explains why $D_{F3} \approx 0.29$ here—slightly lower than $D_{F3} \approx 0.32$ in the single FFC systems (simulation: 0.22 vs. 0.26). Energy concentrates primarily in the NFC (Fig. 4b bottom panel), which exhibits broad-spectrum $|u_z|$ profiles in Supplementary Fig. 12. The computed fields at 1.955 MHz (D_{F3}, top panel in Fig. 4c) reveal a strong FFC u_z (bottom panel), differing slightly from Fig. 4b. Introducing material loss α (Fig. 4d top panel), peaks P_{F3} (1.955 MHz) and P_{N3} (1.966 MHz) persist (Fig. 4d middle/bottom). Supplementary Figs. 13–14 show displacements versus α, which display peaks in $|u_z|$ (P_{N1}-P_{N4}/P_{F1}-P_{F4}) decreasing with α, whereas dips like D_{F2}/D_{F4} exhibit V-shaped profiles with distinct minima.

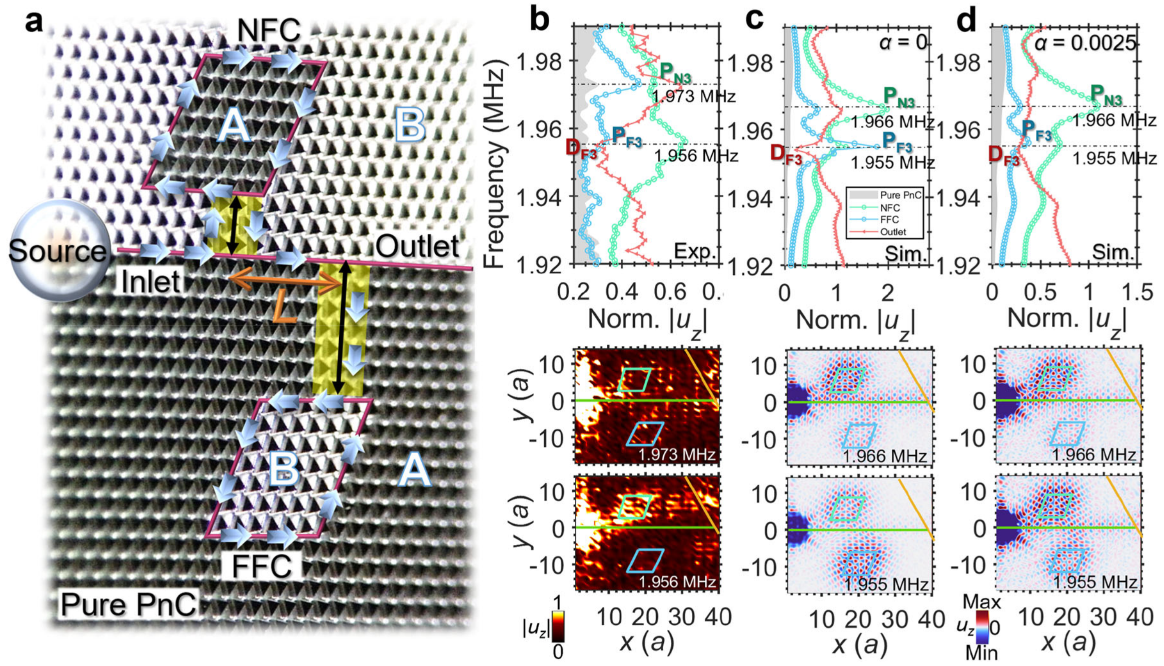

Fig. 4 | Enhanced coupling and sympathetic resonances in the dual-cavity waveguide system. a Optical micrograph of the dual-cavity waveguide phonon system with near-field cavity (NFC, $D = 3 \times 0.866a$) and far-field cavity (FFC, $D = 7 \times 0.866a$) coupled to the same waveguide via Dirac strips. The inter-cavity distance $L = 7a$. **b** Top: Measured normalized $|u_z|$ at the Pure PnC, NFC, FFC, and Outlet positions, excited by a circular pulsed source centered at $f_c = 1.9$ MHz. Bottom: $|u_z|$ distributions at 1.956 MHz (P_{F3}) and 1.973 MHz (P_{N3}). (F/N denotes FFC/NFC; i is the peak/dip index.) The thin lines mark waveguide boundaries and cavity paths. **c**, **d** Simulated spectra corresponding to (**b**) obtained with material loss $\alpha = 0$ (**c**) and $\alpha = 0.0025$ (**d**).

Besides material loss, bulk waves are visible beyond the cavities in the wave field of Fig. 4b. As with the FFC circuit, experimental characterization of the full wave field along the ring paths is limited by our ultrasonic pulses of finite duration and power. Simulations capture the ideal wave-field behaviour, whereas experiments remain essential to reveal the main factors influencing wave propagation.

Space-controlled sympathetic resonances

We now modulate inter-cavity coupling by adjusting the horizontal distance L from $-7a$ to $16a$, probing how sympathetic resonance affects transmission dips. The simulated normalized $|u_z|$ at three positions is displayed in Fig. 5a: waveguide Outlet (left), NFC (middle), and FFC (right), without material loss. Four bright bars labeled P_{N1}-P_{N4} emerge in the NFC panel, whereas four bars P_{F1}-P_{F4} appear in the FFC panel. Frequencies for P_{N1}-P_{N4} and P_{F1}-P_{F4} match their isolated cavity counterparts.

Energy exchange between cavities is evidenced by weak bright bars in the NFC at the frequencies corresponding to P_{F1}-P_{F4} (e.g., dashed lines at P_{F2}/P_{F3}) and in the FFC at the frequencies corresponding to P_{N1}-P_{N4}. The outlet panel shows dark bars (D_{F1}-D_{F4}, D_{N4}) corresponding to resonances (P_{F1}-P_{F4}, P_{N4}).

We now analyze the dual-cavity energy exchange mechanism using governing equations of motion. Let $P_{Fj'}$ denote FFC peaks and $D_{Fj'}$ denote outlet dips for the single cavity circuit and $P_{Fj''}$ and $D_{Fj''}$ denote the analogous quantities for the dual-cavity circuit ($j = 1,2,3,4$). Without material losses, the outlet dips in the dual-cavity circuit are given by $D_{Fj''} = D_{Fj'} \times P_{Fj'}/P_{Fj''}\left(1 + P_{FNj''}\right)$ (see theory). According to this formula, theoretically predicted results (square markers) for D_{F2}-D_{F4} are consistent with direct simulations (solid lines) from the dual-cavity model (Fig. 5b). Relevant results for D_{F1} appear in Supplementary Fig. 15.

In the expression for $D_{Fj''}$, the term $P_{FNj''}$ ($j = 1, 2, 3, 4$) in the denominator refers to the inter-cavity coupling, and serves to reduce the value of $D_{Fj'}$. This system therefore cannot be expressed in terms of the linear overlap of two individual cavities. When L is close to 0, the value of $P_{Fj''}$ is markedly reduced, e.g., for P_{F1}-P_{F3}, which leads to a large value of $D_{Fj''}$ for D_{F1}-D_{F3}, so that critical coupling no longer occurs. Enhanced transmission is not the sole outcome in dual-cavity systems; alternative configurations exist, such as double-sided pillars that broaden plate wave band gaps[34]. Here, the small value of $P_{Fj''}$ when $L \approx 0$ relates to valley PnC properties, such as valley-selected routing paths[24].

The discovery of energy exchange between dual cavities is not only crucial for the emergence of non-Hermitian phenomena, but also underpins sympathetic resonance at shared frequencies. Such non-Hermitian behaviour is vital for high-performance sensing and information processing.

Discussion

In conclusion, we have presented an experimental realization of a far-field phonon coupling circuit by means of a Dirac-strip valley metamaterial. The Dirac strip acts as a broadband, direction-selective transport channel whose linear Dirac dispersion suppresses lateral energy spreading and maintains phase coherence over multiple wavelengths. Unlike the background PnCs used here—which support two-dimensional, laterally spreading wave propagation without directional confinement—the Dirac strip provides a quasi-one-dimensional pathway that efficiently channels cavity radiation toward the waveguide and back. This simultaneously achieves robustness, spectral multiplexing, and distance-insensitive waveguide-cavity energy transfer in an on-chip configuration. In addition, by integrating dual cavities in both the near and far fields, we establish deterministic control over non-Hermitian dynamics through material loss and spatial separation, experimentally observing sympathetic resonances.

In the single-cavity waveguide system, adjusting the length of the Dirac strip optimizes the far-field phonon coupling. In the dual-cavity waveguide system, the simultaneous excitation of both cavities

https://doi.org/10.1038/s41467-025-67108-6

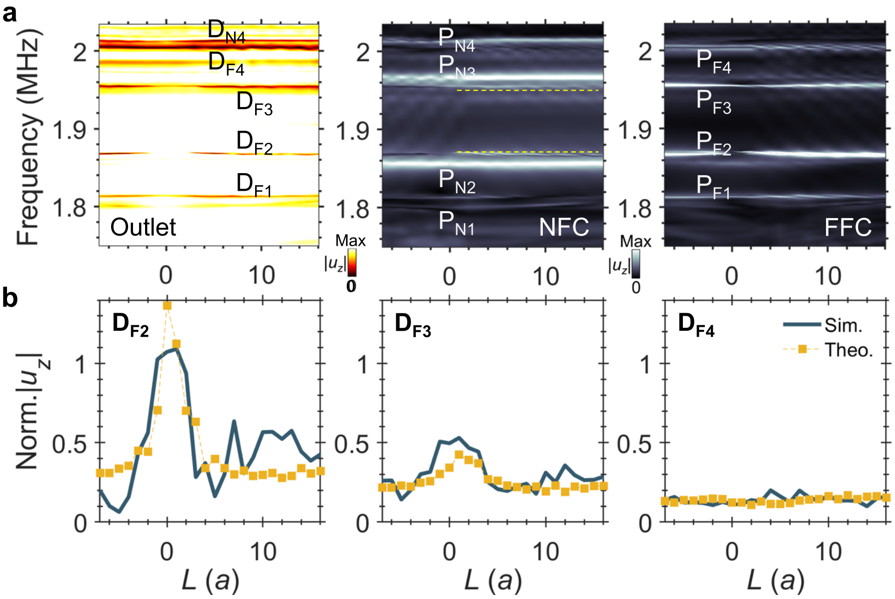

Fig. 5 | Space-controlled coupling and sympathetic resonances in the dual-cavity waveguide system. a Computed normalized $|u_z|$ plotted against frequency and inter-cavity distance L at (left) the Outlet, (middle) the upper edge of the NFC, and (right) the bottom edge of the FFC (positions as in Fig. 4a). Positive L indicates NFC is to the left of FFC; negative $L < 0$ indicates NFC to the right of FFC. **b** Three plots of normalized $|u_z|$ vs. L extracted from D_{F2} to D_{F4} in (**a**), compared with theoretical results calculated from $D_{Fj'} \times P_{Fj'}/P_{Fj''}\left(1+P_{FNj''}\right)$.

produces a pronounced outlet dip compared to the single-cavity system. Furthermore, tuning the inter-cavity distance allows modulation of energy exchange between the dual cavities. The demonstration of far-field coupling over distances up to ~5λ resolves the fundamental challenge of broadband coupling at multiwavelength distances—with direct implications for continuum-mechanical systems requiring spatial decoupling, quantum platforms demanding protected state transfer, photonics, and scalable phononic processors. Furthermore, our platform enables unprecedented pathways for nonlinear wave dynamics[33], analogs of quantum many-body phenomena[35], and topological wave engineering[36,37], directly addressing the escalating need for multi-physical control in integrated wave technologies.

Methods

Experimental methods

The phononic crystal samples were fabricated on 525 µm-thick silicon wafers using lithography and dry etching techniques. During lithography, a 6 µm photoresist layer was applied as an etching mask to protect unpatterned regions, followed by silicon etching in an LPX-ICP system using a BOSCH process to achieve vertical sidewalls. The BOSCH process alternated between passivation and etching steps, with parameters detailed in Supplementary Table 2. Pillar height control was achieved by timing the etching duration based on pre-calibrated silicon etch rates, and final geometries were optically verified.

For all samples (Fig. 1), the wave propagation was characterized using the piezo-laser ultrasonics for in-situ time-resolved measurement of out-of-plane displacement (Supplementary Fig. 1). The displacement u_z was monitored across a $41a \times 29a$ rectangular region encompassing cavities and waveguides, with spatial sampling intervals of 641 µm (a) along x and 555 µm (0.866a) along y. Time-resolved data enabled derivation of average amplitude at the Inlet, Outlet, FFC, NFC, and Pure PnC regions (Figs. 3a and 4b, Supplementary Figs. 11a and 12a). In addition, we carried out spatial field mapping of $|u_z|$ at the resonant frequencies (Figs. 3b and 4b, Supplementary Figs. 9d, 11b and 12b).

As shown in Supplementary Fig. 1, the experimental setup comprised a Polytec OFV 2570 laser Doppler vibrometer (LDV), a high-speed camera, a RIGOL DG1032z signal generator, a power amplifier, and a Tektronix DPO4102B-L oscilloscope. Silicon samples were mounted on a supporting plate with a spatial positioning precision of ~5 µm. A 5 mm × 1 mm PZT disk was bonded to the back surface of the waveguide inlet using conductive adhesive. A seven-cycle sinusoidal burst signal centered at frequency f_c was generated, amplified, and delivered to the PZT transducer. The number of cycles in the burst was limited to facilitate identification of the incident wave packet as it propagated through the valley metamaterial phononic circuits. The LDV measured the out-of-plane displacement (u_z) within target regions, while the high-speed camera recorded the laser beam position and enabled spatial point selection.

To enhance experimental accuracy, several measures were taken. The input signal power was amplified by ~37 dB to generate strong ultrasonic pulses without damaging the silicon wafer. At each spatial point, 512 scans were averaged to reduce white noise. The sampling frequency was set to f_s = 100 MHz, more than 47 times the target frequency f_c (1.7–2.1 MHz). At each spatial point, signals were recorded with 10^5 data points, corresponding to >1700 periods of $1/f_c$. This allowed fine spectral filtering near f_c with a frequency resolution of ~0.001 MHz. By repeating these procedures, we obtained the amplitude of the out-of-plane displacement across the target region, enabling separation of neighboring TWGMs and dips. These procedures also allowed us to reconstruct animations and spatial segments of wave displacement within the region of interest.

Numerical calculations

We adopt the finite-element method to calculate dispersion in our silicon phononic crystal plates. Elastic constants are taken as C_{11} = 165.6 GPa, C_{12} = 63.9 GPa, C_{44} = 79.5 GPa, and mass density 2.331 g/cm^3[38], with silicon assumed to exhibit near-isotropic behaviour. Unit cell dispersion is computed for rotation angles $\theta = 0°$ and ±20° (Fig. 1c, d).

For topological edge states (TESs), a sandwiched supercell (Supplementary Fig. 5a) comprising 12-layer PnC-B (top), 12-layer PnC-A (middle), and 12-layer PnC-B (bottom) is constructed. Periodic boundary conditions are applied to the supercell B-A-B along the x-direction, whereas continuity conditions are applied along the

y-direction. The results for dispersion (Supplementary Fig. 5b, c) include the displacement ratio $\int |u_z|dV / \int |u_t|dV$ at each frequency, where u_z and u_t denote out-of-plane and total displacements.

Topological whispering gallery modes (TWGMs) are analyzed using a rhombus cavity supercell (side length $7a$) positioned between the inner PnC-B and outer PnC-A (Supplementary Fig. 6a). Periodic boundary conditions are applied to three pairs of parallel sides of the hexagonal supercell, yielding the corresponding eigenfrequencies and displacement fields (Fig. 2b; Supplementary Fig. 6b, c).

Wave propagation simulations for NFC-waveguide, FFC-waveguide (Fig. 2a), and dual-cavity structures (Fig. 4a) replicate experimental dimensions (Fig. 1a). Perfectly matched layers (PMLs) absorb boundary reflections. A circular source applies unit z-axis force over a 2.5 mm radius at the waveguide inlet. Transmission spectra (Figs. 3c and 4c, d, Supplementary Figs. 9a, 11c, 12c and 13) are based on the average $|u_z|$ over $5a$ segments at Inlet, Outlet, FFC, NFC, and unpatterned PnC regions.

In single cavity-waveguide models (Fig. 2a), the Dirac strip length D varies from 1 to 12 layers in 1-layer increments. Frequency- and distance-resolved $|u_z|$ maps (Fig. 2c) are generated from average values over $5a$ segments at the Outlet and FFC. Control simulations replacing Dirac strips with PnC-A appear in Fig. 2d. For dual-cavity models (Fig. 4a), the inter-cavity distance L sweeps from $-7a$ to $16a$ in steps of a, producing frequency-distance $|u_z|$ maps at the Outlet, NFC, and FFC (Fig. 5a).

Theory of the waveguide-cavity dynamics

Single-cavity waveguide system. The waveguide-cavity coupling can be understood from the theoretical model shown in Supplementary Fig. 2. The cavity lies either above or below the waveguide. From theory[39,40], the incident wave S_+ gives rise to a cavity response $\tilde{a}_c = a_c e^{i\omega t}$. Γ and γ are the dissipative and radiative losses of the cavity, respectively.

The governing equation can be expressed as[40–42]

$$\frac{d\tilde{a}_c}{dt} = (i\omega_0 - \Gamma - \gamma)\tilde{a}_c + i\sqrt{\gamma}S_+, \tag{1}$$

where $\omega_0 = 2\pi f_0$ represents the resonant frequency of the cavity. Silicon can be considered to be close to an isotropic material, so, in contrast to previous work[20], material anisotropy is ignored in this equation.

The derivative of $\tilde{a}_c = a_c e^{i\omega t}$ is given by

$$\frac{d\tilde{a}_c}{dt} = \frac{d(a_c e^{i\omega t})}{dt} = \frac{da_c}{dt}e^{i\omega t} + a_c\frac{d(e^{i\omega t})}{dt} = \frac{da_c}{dt}e^{i\omega t} + i\omega\tilde{a}_c. \tag{2}$$

When the amplitude remains constant, $da_c/dt = 0$, yielding

$$\frac{d\tilde{a}_c}{dt} = i\omega\tilde{a}_c. \tag{3}$$

Substituting Eq. (3) into Eq. (1) yields

$$\tilde{a}_c = \frac{i\sqrt{\gamma}}{i(\omega - \omega_0) + \Gamma + \gamma}S_+. \tag{4}$$

The relationship between the input wave and the output wave S_- can be written as

$$S_- = S_+ + i\sqrt{\gamma}\tilde{a}_c. \tag{5}$$

Combining Eqs. (4) and (5), the transmission coefficient of the system can be expressed in the form

$$T = \left|\frac{S_-}{S_+}\right| = \left|\frac{S_+ + i\sqrt{\gamma}\tilde{a}_c}{S_+}\right| = \left|1 + \frac{-\gamma}{i(\omega - \omega_0) + \Gamma + \gamma}\right|. \tag{6}$$

According to Eq. (6), one obtains

$$\begin{cases} \Delta\omega = 2(\Gamma + \gamma) \\ T_{\min} = \frac{\Gamma}{\Gamma + \gamma} \end{cases} \tag{7}$$

where $T_{\min}$ is the minimum value around ω_0, and $\Delta\omega$ represents the angular frequency corresponding to the full width at half of $T_{\min}$. These two parameters can be obtained from the numerically calculated spectrum. The substitution of $T_{\min}$ and $\Delta\omega$ into Eq. (7) yields the dissipative loss Γ and radiation loss γ. Then, by use of Eq. (6), one can plot the fitted transmission profile around ω_0. Critical coupling occurs when $\Gamma = \gamma$, whereas $\Gamma < \gamma$ (or $\Gamma > \gamma$) indicates over- (or under-) coupling.

Dual-cavity waveguide system. The theoretical model of the dual-cavity waveguide system is illustrated in Supplementary Fig. 3. Each cavity includes dissipative loss Γ_j and radiative loss γ_j (j = F, N). Since the two cavities are inserted on separate sides of the waveguide, direct cavity-to-cavity coupling is prohibited. The quantity φ is the accumulated phase difference along the waveguide path.

For an incident wave S_+, the governing equation for the two-cavity system ($\tilde{a}_N = a_N e^{i\omega t}$ and $\tilde{a}_F = a_F e^{i\omega t}$) can be written as[43,44]

$$\frac{d\tilde{a}_N}{dt} = (i\omega_N - \Gamma_N - \gamma_N)\tilde{a}_N + i\sqrt{\gamma_N}(S_+ + i\sqrt{\gamma_F}e^{-i\varphi}\tilde{a}_F), \tag{8}$$

$$\frac{d\tilde{a}_F}{dt} = (i\omega_F - \Gamma_F - \gamma_F)\tilde{a}_F + i\sqrt{\gamma_F}(S_+ e^{-i\varphi} + i\sqrt{\gamma_N}e^{-i\varphi}\tilde{a}_N), \tag{9}$$

where ω_N and ω_F are the resonance frequencies of the near- and far-field cavities, respectively. Direct cavity-to-cavity coupling is ignored. On the right-hand side of Eq. (8), the 2nd term includes two contributions: one from the coupling between the upper cavity (a_N) and the incoming waves, the other from the radiated field from the lower cavity (a_F). Equation (9) can be understood in a similar way.

The transmission coefficient for the system is given by

$$\begin{aligned} T &= \left|\frac{S_-}{S_+}\right| = \left|\frac{(S_+ + i\sqrt{\gamma_N}\tilde{a}_N)e^{-i\varphi} + i\sqrt{\gamma_F}\tilde{a}_F}{S_+}\right| \\ &= \left|e^{-i\varphi} + i(e^{-i\varphi}\sqrt{\gamma_N}\tilde{a}_N/S_+ + \sqrt{\gamma_F}\tilde{a}_F/S_+)\right|. \end{aligned} \tag{10}$$

By fitting the simulated transmission profile, we can obtain parameters including Γ_j and γ_j (j = F, N). Substituting them into Eqs. (8) and (9) yields $\tilde{a}_N$ and $\tilde{a}_F$. One can then plot the theoretically fitted profile of transmission from Eq. (10).

When $\omega = \omega_F$,

$$T = \left|e^{-i\varphi} + i(e^{-i\varphi}\sqrt{\gamma_N}a_N/S_+ + \sqrt{\gamma_F}a_F/S_+)\right| = D_{Fj}''. \tag{11}$$

Specifically,

$$D_{Fj}'' = \left|\frac{e^{i\varphi}([i(\omega - \omega_F) + \Gamma_F + \gamma_F] - \gamma_F)([i(\omega - \omega_N) + \Gamma_N + \gamma_N] - \gamma_N)}{e^{2i\varphi}[i(\omega - \omega_N) + \Gamma_N + \gamma_N][i(\omega - \omega_F) + \Gamma_F + \gamma_F] - \gamma_F\gamma_N}\right|, \tag{12}$$

where the D_{Fj}'' is the outlet dip in the dual-cavity circuit.

Combining Eqs. (8) and (9), the amplitudes for the NFC and FFC are given by

$$\left|\frac{\tilde{a}_N}{S_+}\right| = \left|\frac{i\sqrt{\gamma_N}\{e^{2i\varphi}[i(\omega-\omega_F)+\Gamma_F+\gamma_F]-\gamma_F\}}{e^{2i\varphi}[i(\omega-\omega_N)+\Gamma_N+\gamma_N][i(\omega-\omega_F)+\Gamma_F+\gamma_F]-\gamma_F\gamma_N}\right|, \quad (13)$$

$$\left|\frac{\tilde{a}_F}{S_+}\right| = \left|\frac{i\sqrt{\gamma_F}\{e^{i\varphi}[i(\omega-\omega_F)+\Gamma_F+\gamma_F]-e^{i\varphi}\gamma_N\}}{e^{2i\varphi}[i(\omega-\omega_N)+\Gamma_N+\gamma_N][i(\omega-\omega_F)+\Gamma_F+\gamma_F]-\gamma_F\gamma_N}\right| = P_{Fj}'', \quad (14)$$

where P_{Fj}'' refers to the amplitude peak for the FFC ($\omega = \omega_F$) in the dual-cavity circuit.

As a comparison, consider again the single FFC-waveguide model and set $\omega = \omega_0 = \omega_F$. According to Eqs. (4) and (6), the outlet dip D_{Fj}' and the FFC peak P_{Fj}' can be derived as follows:

$$D_{Fj}' = \left|1 - \frac{\gamma_F}{\Gamma_F+\gamma_F}\right|, \quad (15)$$

$$P_{Fj}' = \left|\frac{i\sqrt{\gamma_F}}{\Gamma_F+\gamma_F}\right|. \quad (16)$$

Combining Eqs. (12), (14), (15) and (16),

$$\frac{D_{Fj}''P_{Fj}''}{D_{Fj}'P_{Fj}'} = \left|\frac{e^{2i\varphi}[i(\omega-\omega_F)+\Gamma_F+\gamma_F]^2\{[i(\omega-\omega_N)+\Gamma_N+\gamma_N]-\gamma_N\}^2}{\{e^{2i\varphi}[i(\omega-\omega_N)+\Gamma_N+\gamma_N][i(\omega-\omega_F)+\Gamma_F+\gamma_F]-\gamma_F\gamma_N\}^2}\right|. \quad (17)$$

If the two cavities possess different resonance frequencies ($\omega_N \neq \omega_F$), then when $\omega = \omega_F$,

$$i(\omega-\omega_F)+\Gamma_F+\gamma_F|_{\omega=\omega_F} = \Gamma_F+\gamma_F, \quad (18)$$

and the term $i(\omega-\omega_N)+\Gamma_N+\gamma_N$ can be seen to contain extra terms compared to $\Gamma_F+\gamma_F$:

$$i(\omega-\omega_N)+\Gamma_N+\gamma_N = \Gamma_F+\mathrm{d}\Gamma+\gamma_F+\mathrm{d}\gamma+i\mathrm{d}\omega. \quad (19)$$

One can rewrite Eq. (17) as

$$\frac{D_{Fj}'P_{Fj}'}{D_{Fj}'P_{Fj}''} = \left|\frac{e^{-2i\varphi}(\gamma_F{}^2-e^{2i\varphi}(\gamma_F+\Gamma_F)^2)^2}{\Gamma_F{}^2(\gamma_F+\Gamma_F)^2}\right| + O(\mathrm{d}\Gamma,\mathrm{d}\gamma,\mathrm{d}\omega), \quad (20)$$

and similarly for Eq. (20):

$$\frac{D_{Fj}'P_{Fj}'}{D_{Fj}''P_{Fj}''} = \frac{(2\gamma_F+\Gamma_F)^2}{(\gamma_F+\Gamma_F)^2} + O(\mathrm{d}\Gamma,\mathrm{d}\gamma,\mathrm{d}\omega,\varphi) = 1 + P_{FNj}'', \quad (21)$$

where

$$P_{FNj}'' = \frac{2\gamma_F}{\gamma_F+\Gamma_F} + \left(\frac{\gamma_F}{\gamma_F+\Gamma_F}\right)^2 + O(\mathrm{d}\Gamma,\mathrm{d}\gamma,\mathrm{d}\omega,\varphi). \quad (22)$$

Finally, the outlet dip in the dual-cavity circuit can be expressed as

$$D_{Fj}'' = D_{Fj}'\frac{P_{Fj}'}{P_{Fj}''(1+P_{FNj}'')}. \quad (23)$$

Based on Eq. (12), the outlet dip is governed by Γ_j and γ_j (j = F, N). Equation (23) shows that the inter-cavity coupling affects the outlet dip D_{Fj}'', which can be seen in the term $(1+P_{FNj}'')$.

Data availability

The data that support the findings of this study are available from the corresponding author upon request.

References

1. Rayleigh, L. The problem of the whispering gallery. *Philos. Mag.* **20**, 1001–1004 (1910).
2. Xu, X. et al. Acoustofluidic tweezers via ring resonance. *Sci. Adv.* **10**, eads2654 (2024).
3. Mezil, S. et al. Active chiral control of GHz acoustic whispering-gallery modes. *Appl. Phys. Lett.* **111**, 144103 (2017).
4. Armani, D. K., Kippenberg, T. J., Spillane, S. M. & Vahala, K. J. Ultra-high-Q toroid microcavity on a chip. *Nature* **421**, 922–925 (2003).
5. Henke, J. et al. Integrated photonics enables continuous-beam electron phase modulation. *Nature* **600**, 653–658 (2021).
6. Bahari, B. et al. Nonreciprocal lasing in topological cavities of arbitrary geometries. *Science* **358**, 636–640 (2017).
7. Wang, Y., Zeng, S., Humbert, G. & Ho, H. P. Microfluidic whispering gallery mode optical sensors for biological applications. *Laser Photonics Rev.* **14**, 2000135 (2020).
8. Hu, Y. et al. High-efficiency and broadband on-chip electro-optic frequency comb generators. *Nat. Photonics* **16**, 679–685 (2022).
9. Chen, W., Kaya, O. S., Zhao, G., Wiersig, J. & Yang, L. Exceptional points enhance sensing in an optical microcavity. *Nature* **548**, 192–196 (2017).
10. Miri, M. & Alù, A. Exceptional points in optics and photonics. *Science* **363**, eaar7709 (2019).
11. Peng, B. et al. Loss-induced suppression and revival of lasing. *Science* **346**, 328–332 (2014).
12. Li, A. et al. Exceptional points and non-Hermitian photonics at the nanoscale. *Nat. Nanotechnol.* **18**, 706–720 (2023).
13. Patil, Y. S. S. et al. Measuring the knot of non-Hermitian degeneracies and non-commuting braids. *Nature* **607**, 271–275 (2022).
14. Parto, M., Leefmans, C., Williams, J., Nori, F. & Marandi, A. Non-Abelian effects in dissipative photonic topological lattices. *Nat. Commun.* **14**, 1440 (2023).
15. Bergholtz, E. J., Budich, J. C. & Kunst, F. K. Exceptional topology of non-Hermitian systems. *Rev. Mod. Phys.* **93**, 01505 (2021).
16. El-Ganainy, R. et al. Non-Hermitian physics and PT symmetry. *Nat. Phys.* **14**, 11–19 (2018).
17. Parappurath, N., Alpeggiani, F., Kuipers, L. & Verhagen, E. Direct observation of topological edge states in silicon photonic crystals: spin, dispersion, and chiral routing. *Sci. Adv.* **6**, eaaw4137 (2020).
18. Barik, S., Karasahin, A., Flower, C. & Cai, T. A topological quantum optics interface. *Science* **359**, 666–668 (2018).
19. Yu, S. et al. Critical couplings in topological-insulator waveguide-resonator systems observed in elastic waves. *Natl Sci. Rev.* **8**, nwaa262 (2021).
20. Hatanaka, D. et al. Valley pseudospin polarized evanescent coupling between microwave ring resonator and waveguide in phononic topological insulators. *Nano Lett.* **24**, 5570–5577 (2024).
21. Huang, Y. et al. Parity-frequency-space elastic spin control of wave routing in topological phononic circuits. *Adv. Sci.* **11**, 2404839 (2024).
22. Li, J., Deng, C., Zhang, K., Lu, Q. & Yang, H. Higher-order topological states in dual-band valley sonic crystals. *Appl. Phys. Lett.* **123**, 253101 (2023).
23. Wu, X. et al. Topological corner modes induced by Dirac vortices in arbitrary geometry. *Phys. Rev. Lett.* **126**, 226802 (2021).
24. Yan, M. et al. On-chip valley topological materials for elastic wave manipulation. *Nat. Mater.* **17**, 993–998 (2018).
25. Zhang, Q. et al. Gigahertz topological valley Hall effect in nanoelectromechanical phononic crystals. *Nat. Electron.* **5**, 157–163 (2022).

26. Hu, B. et al. Non-Hermitian topological whispering gallery. *Nature* **597**, 655–659 (2021).
27. Zeng, Y. et al. Electrically pumped topological laser with valley edge modes. *Nature* **578**, 246–250 (2020).
28. Jia, R. et al. On-chip active supercoupled topological cavity. *Adv. Mater.* **37**, 2419261 (2025).
29. Zhao, J. et al. Elastic valley spin controlled chiral coupling in topological valley phononic crystals. *Phys. Rev. Lett.* **129**, 275501 (2022).
30. Wang, M. et al. Valley-locked waveguide transport in acoustic heterostructures. *Nat. Commun.* **11**, 3000 (2020).
31. Zhang, Z., Lu, M. & Chen, Y. Observation of free-boundary-induced chiral anomaly bulk states in elastic twisted kagome metamaterials. *Phys. Rev. Lett.* **132**, 086302 (2024).
32. Rosiek, C. A. et al. Observation of strong backscattering in valley-Hall photonic topological interface modes. *Nat. Photonics* **17**, 386–392 (2023).
33. Shim, S., Imboden, M. & Mohanty, P. Synchronized oscillation in coupled nanomechanical oscillators. *Science* **316**, 95–99 (2007).
34. Badreddine, A. M. & Oudich, M. Enlargement of a locally resonant sonic band gap by using double-sides stubbed phononic plates. *Appl. Phys. Lett.* **100**, 123506 (2012).
35. Liška, V. et al. PT-like phase transition and limit cycle oscillations in non-reciprocally coupled optomechanical oscillators levitated in vacuum. *Nat. Phys.* **20**, 1622–1628 (2024).
36. Lu, X., McClung, A. & Srinivasan, K. High-Q slow light and its localization in a photonic crystal microring. *Nat. Photonics* **16**, 66–71 (2021).
37. Zhang, Z. et al. Tunable topological charge vortex microlaser. *Science* **368**, 760–763 (2020).
38. Zhao, J., Bonello, B. & Boyko, O. Focusing of the lowest-order antisymmetric Lamb mode behind a gradient-index acoustic metalens with local resonators. *Phys. Rev. B* **93**, 174306 (2016).
39. Righini, G. C. et al. Whispering gallery mode microresonators: Fundamentals and applications. *La Riv. del. Nuovo Cim.* **34**, 435–488 (2011).
40. Yariv, A. Critical coupling and its control in optical waveguide-ring resonator systems. *IEEE Photonics Tech. Lett.* **14**, 483–485 (2002).
41. Yariv, A. Universal relations for coupling of optical power-betweenmicroresonators and dielectric waveguides. *Electron. Lett.* **36**, 321–322 (2000).
42. Haus, H. A. *Waves and Fields in Optoelectronics* Vol. 464 (Prentice Hall, 1984).
43. Tan, W., Sun, Y., Wang, Z. & Chen, H. Manipulating electromagnetic responses of metal wires at the deep subwavelength scale via both near- and far-field couplings. *Appl. Phys. Lett.* **104**, 091107 (2014).
44. Kekatpure, R. D., Barnard, E. S., Cai, W. & Brongersma, M. L. Phase-coupled plasmon-induced transparency. *Phys. Rev. Lett.* **104**, 243902 (2010).

Acknowledgements

This work was supported by the National Natural Science Foundation of China (Grants No. 12172256, 12302115, 12272269, 11972257, 52027816). It was also supported by Shanghai Leading Talent Program of Eastern Talent Plan. J. Z. would like to acknowledge Dr. Haoyun Tu's help with the experiment.

Author contributions

Yao H., Weitao Y. and Jinfeng Z. designed the sample structures. Qi W. and Jia Z. fabricated the experimental samples. Yao H. performed the experimental measurements, carried out the numerical simulations, analyzed and visualized all data, and wrote the first draft of the manuscript. Weitao Y. and Jinfeng Z. helped check the simulation results. Weitao Y., Yao H., Zhiwei G. and Jinfeng Z. derived the theory. Jinfeng Z. conceived the idea, designed the experimental setups and analyzed the initial experimental data. Yuxuan Z. and Yongdong P. assisted in the experiments. Yueting Z., Zheng Z. and Oliver B. Wright helped in the derivation of the theory. Jinfeng. Z., Weitao Y., Yueting Z., Yongdong P. and Zhiwei G. conceived the project. Oliver B. Wright constructed the final version of the manuscript. All the authors contributed to discussions, interpretation of the data, and the writing process.

Competing interests

The authors declare no competing interests.

Additional information

Supplementary information The online version contains supplementary material available at https://doi.org/10.1038/s41467-025-67108-6.

Correspondence and requests for materials should be addressed to Jinfeng Zhao.

Peer review information *Nature Communications* thanks the anonymous reviewers for their contribution to the peer review of this work. A peer review file is available.

Reprints and permissions information is available at http://www.nature.com/reprints

Publisher's note Springer Nature remains neutral with regard to jurisdictional claims in published maps and institutional affiliations.

Open Access This article is licensed under a Creative Commons Attribution 4.0 International License, which permits use, sharing, adaptation, distribution and reproduction in any medium or format, as long as you give appropriate credit to the original author(s) and the source, provide a link to the Creative Commons licence, and indicate if changes were made. The images or other third party material in this article are included in the article's Creative Commons licence, unless indicated otherwise in a credit line to the material. If material is not included in the article's Creative Commons licence and your intended use is not permitted by statutory regulation or exceeds the permitted use, you will need to obtain permission directly from the copyright holder. To view a copy of this licence, visit http://creativecommons.org/licenses/by/4.0/.

© The Author(s) 2025
